# Supplementary material for: In vivo assessment of neurodegeneration in Spinocerebellar Ataxia type 7
Source: Neuroimage Clin. 2021 Jan 19;29:102561. doi: 10.1016/j.nicl.2021.102561 (PMC7848632; doi:10.1016/j.nicl.2021.102561)
Supplement: Supplementary data 1 [file mmc1.docx]

**Supplementary Materials 1 of 2**

**Contents**

**S1: VBM, MD, and FA group comparisons**

**Figure S1.1:** VBM group comparison

**Figure S1.2:** MD group comparison

**Figure S1.3:** FA group comparison

**S2: Group comparison effect size maps**

**Figure S2.1:** DTBM (logJ) group comparison effect size map

**Figure S2.2:** pVF group comparison effect size map

**Figure S2.3:** pMD group comparison effect size map

**Figure S2.4:** pFA group comparison effect size map

**Figure S2.5:** VBM group comparison effect size map

**Figure S2.6:** MD group comparison effect size map

**Figure S2.7:** FA group comparison effect size map

**S3: VBM, MD, and FA group comparison tables of most affected regions**

**S3.1:** VBM table

**Table S3.1.1:** VBM FreeSurfer GM atlas

**S3.2:** MD tables

**Table S3.2.1:** MD FreeSurfer GM atlas

**Table S3.2.2:** MD ICBM-DTI-81 WM atlas

**S3.3:** FA tables

**Table S3.3.1:** FA FreeSurfer GM atlas

**Table S3.3.2:** FA ICBM-DTI-81 WM atlas

**S4: Correlation analysis supplementary material**

**Figure S4.1:** Correlations between imaging metrics and SARA score

**Figure S4.2:** Correlations of single compartment diffusivity metrics (MD and FA) and dual compartment diffusivity metrics (pMD and pFA) with the parenchymal VF

**Figure S4.3:** Correlations of whole brain pFA and cerebellar pVF with the SARA score with asymptomatic patient included

**Figure S4.4:** Correlations between imaging metrics and SARA score with asymptomatic patient included

**S1 VBM, MD, and FA group comparisons**

**Figure S1.1:** VBM group comparison

**
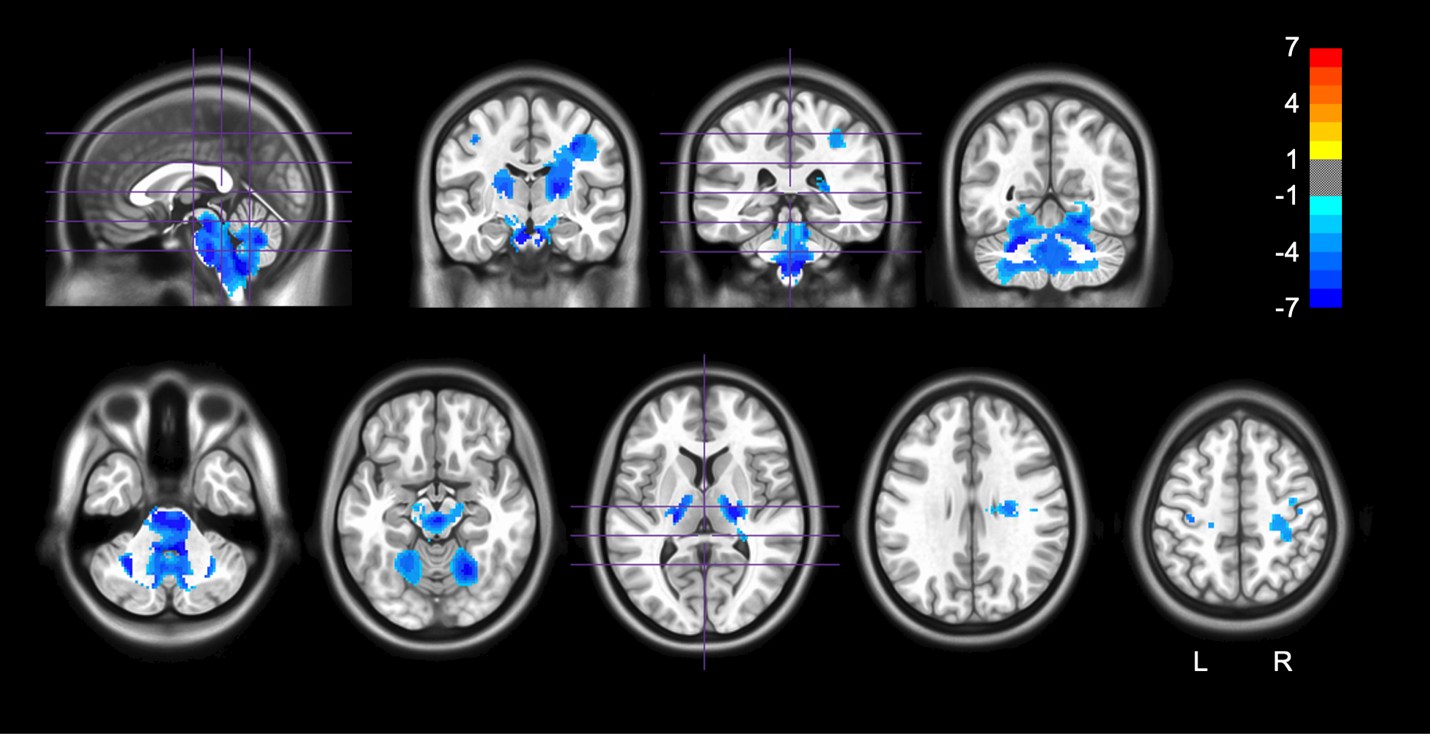
**

Figure S1.1: Regions in which GM volume was significantly different between HVs and SCA7 (*p*<0.05, FWE corrected). Values shown are the *t*-statistic. Blue indicates a lower GM volume in SCA7 patients versus HVs. Orange indicates a higher GM volume. Results are overlaid on the MNI ICBM 2009c Nonlinear Asymmetric template. See Table S3.1.1 for a list of regions.

**Figure S1.2:** MD group comparison

**
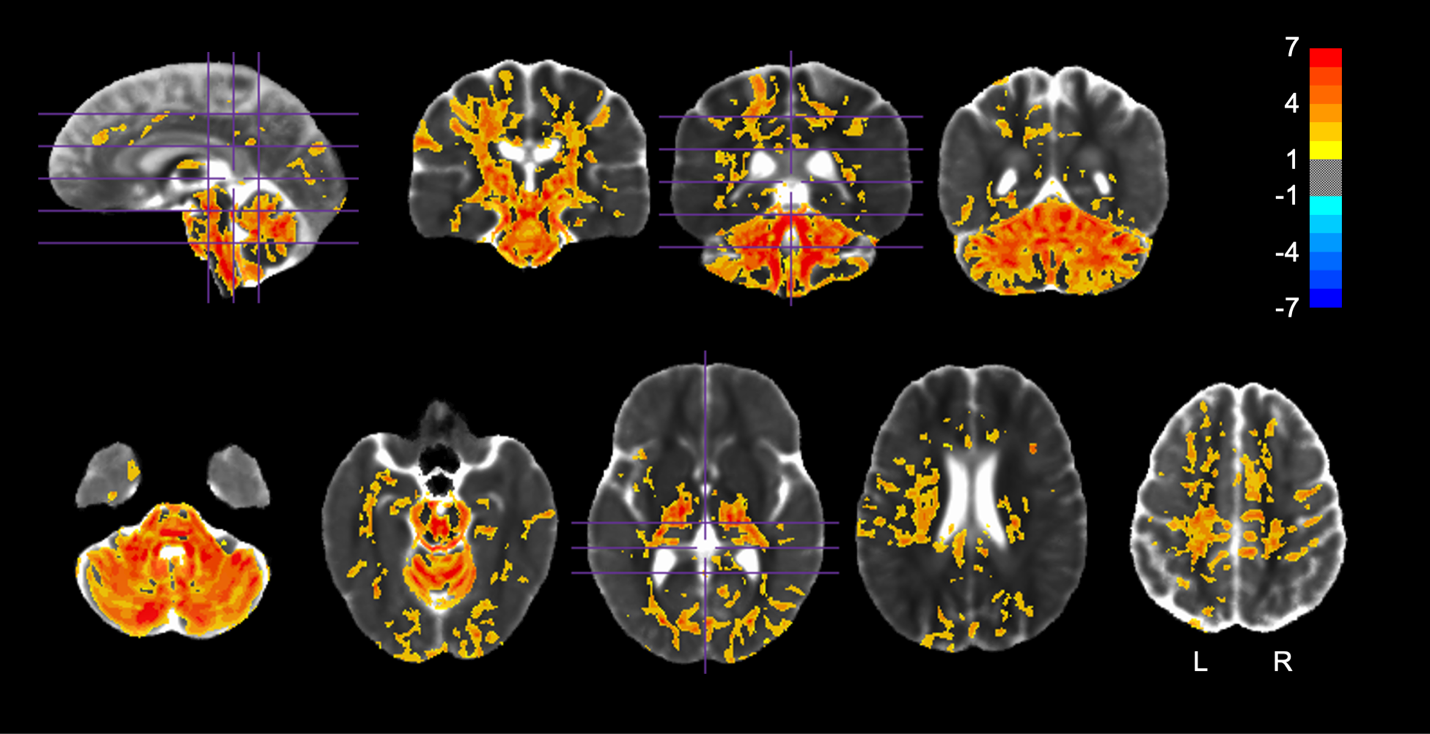
**

Figure S1.2: Regions in which MD was significantly different between HVs and SCA7 (*p*<0.05, FWE corrected). Values shown are the *t*-statistic. Orange indicates a higher MD in SCA7 patients versus HVs. Blue indicates a lower MD. Results are overlaid on the HV diffusion tensor template. See Table S3.2.1 and Table S3.2.2 for a list of regions.

**Figure S1.3:** FA group comparison

**
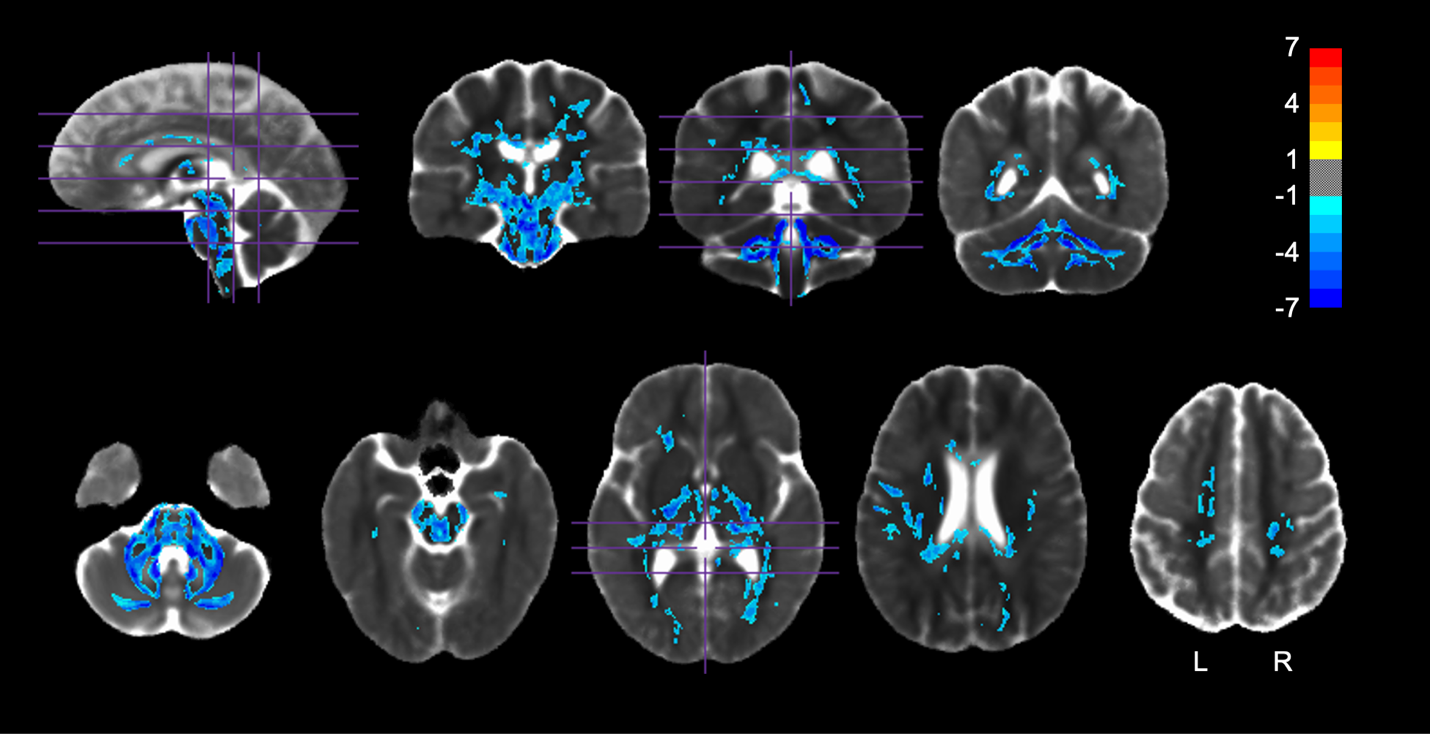
**

Figure S1.3: Regions in FA was significantly different between HVs and SCA7 (*p*<0.05, FWE corrected). Values shown are the *t*-statistic. Blue indicates a lower FA in SCA7 patients versus HVs. Orange indicates a higher FA. Results are overlaid on the HV diffusion tensor template. See Table S3.3.1 and Table S3.3.2 for a list of regions.

**S2 Group comparison effect size maps**

**Figure S2.1 DTBM (logJ) group comparison effect size map**

*
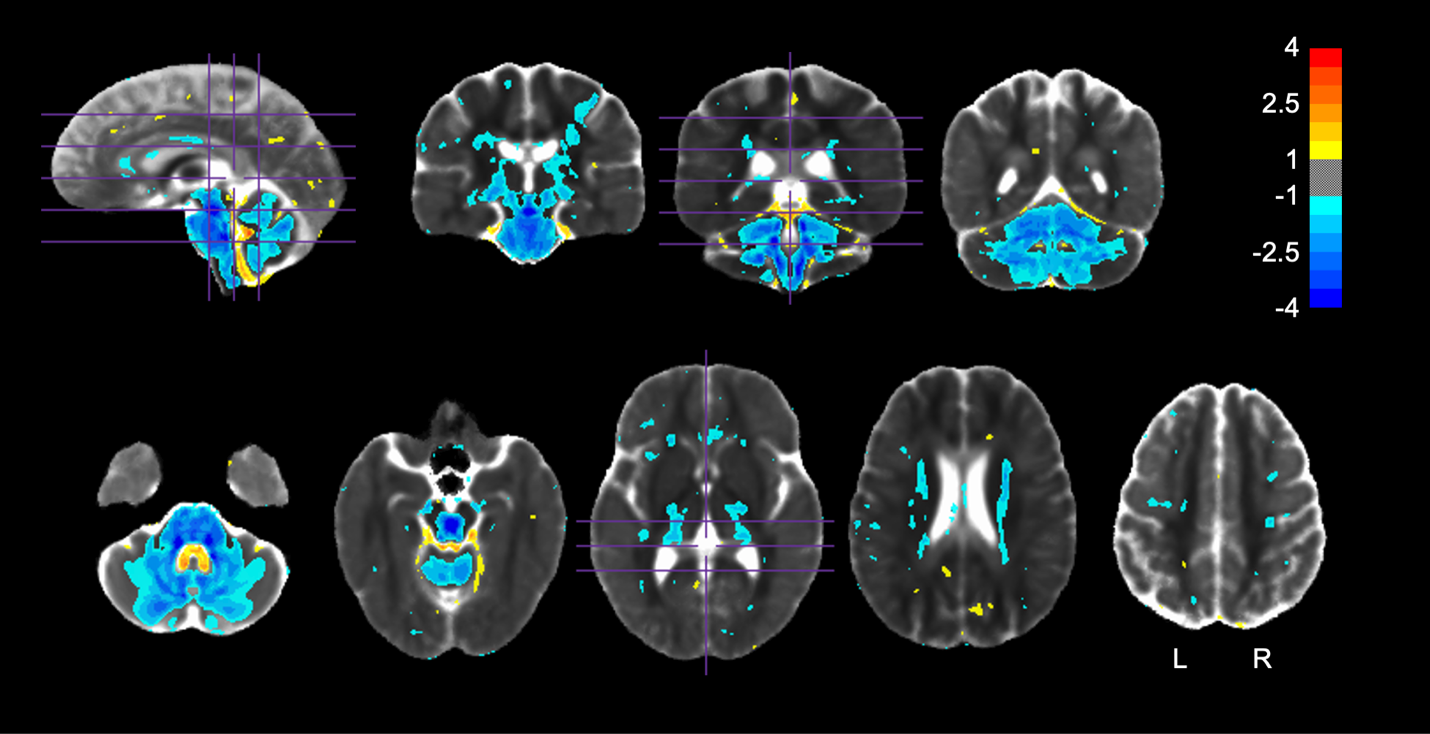
*

Figure S2.1: Regions in which the absolute value of Hedge’s *g* for the logJ HV versus SCA7 comparison exceeded 1. Values shown are Hedge’s *g*. Blue indicates a lower volume (lower logJ) in SCA7 patients versus HVs. Orange indicates a higher volume in SCA7 patients versus HVs. Results are overlaid on the HV diffusion tensor template.

**Figure S2.2 pVF group comparison effect size map**


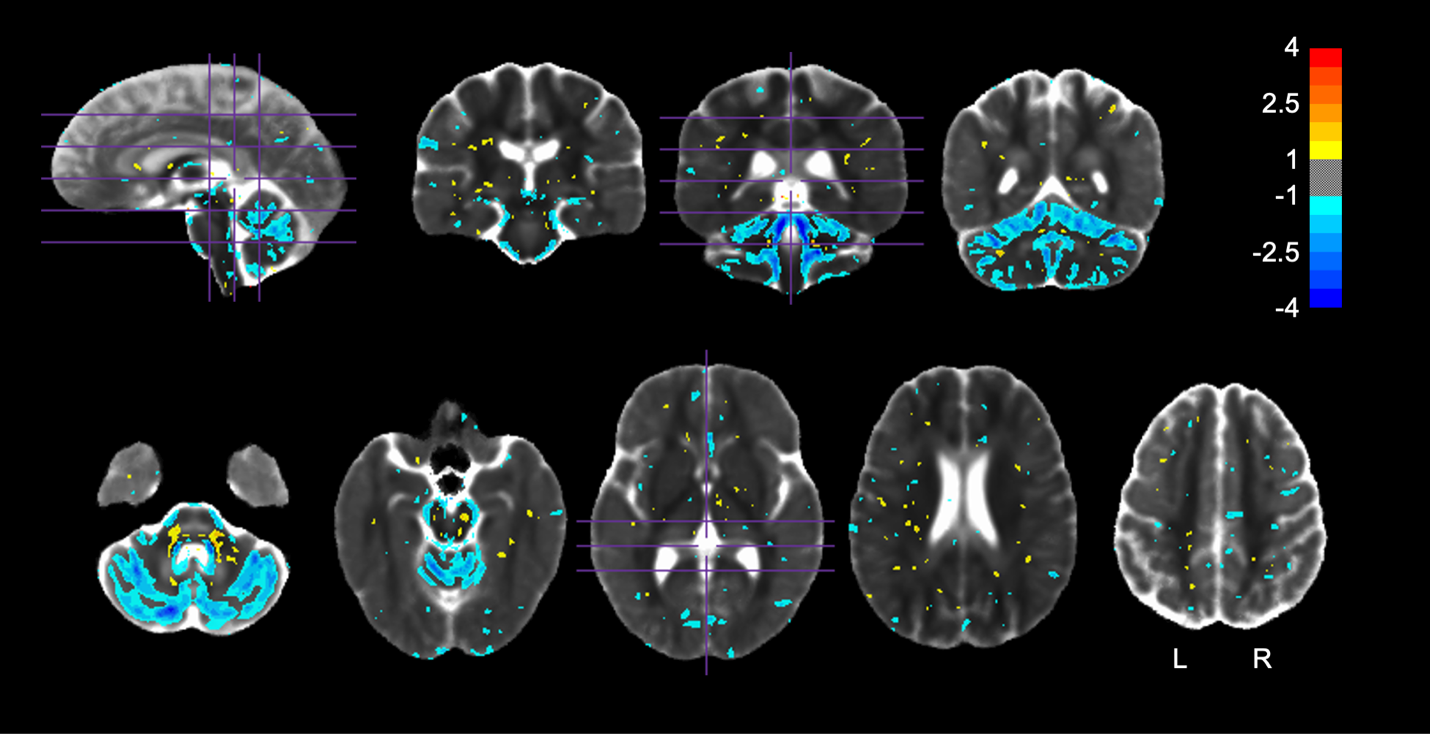


Figure S2.2: Regions in which the absolute value of Hedge’s *g* for the pVF HV versus SCA7 comparison exceeded 1. Values shown are Hedge’s *g*. Blue indicates a lower pVF in SCA7 patients versus HVs. Orange indicates a higher pVF in SCA7 patients versus HVs. Results are overlaid on the HV diffusion tensor template.

**Figure S2.3 pMD group comparison effect size map**


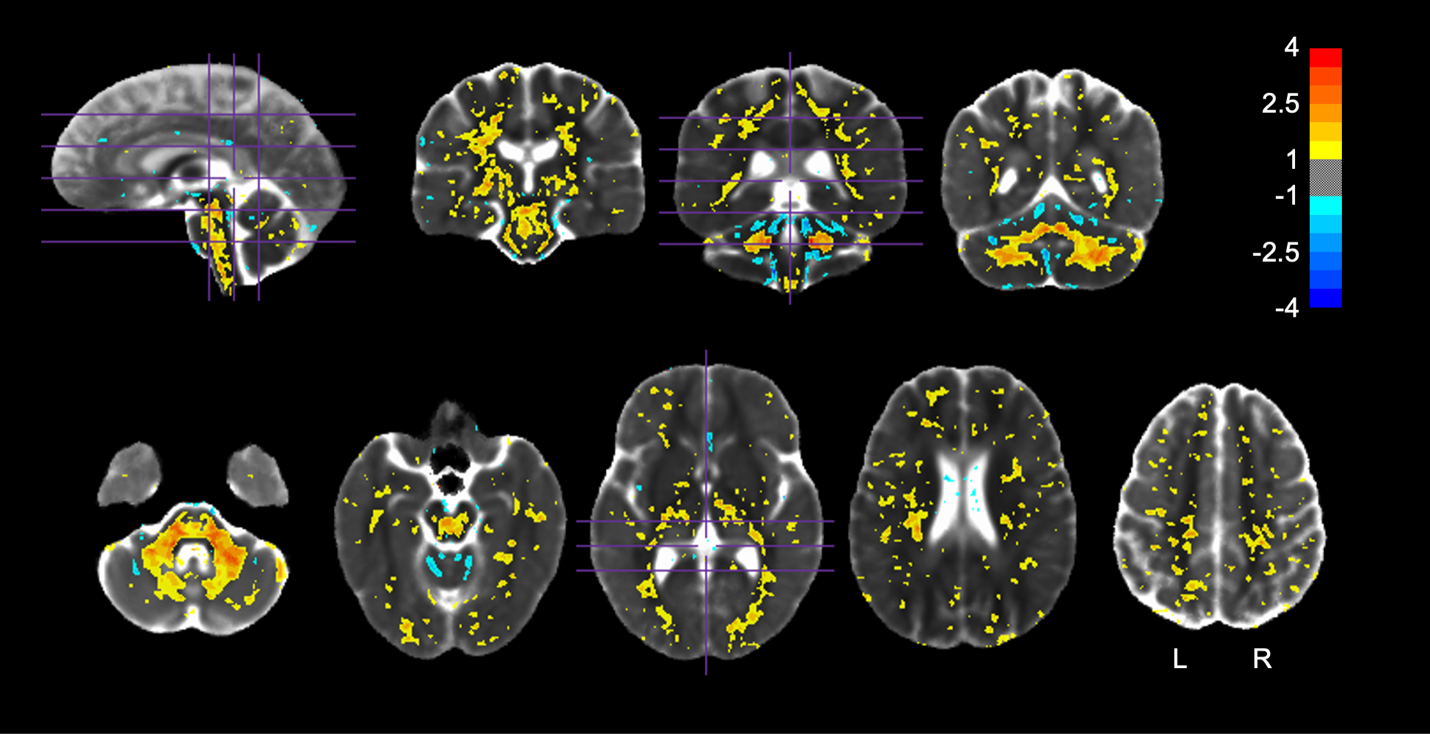


Figure S2.3: Regions in which the absolute value of Hedge’s *g* for the pMD HV versus SCA7 comparison exceeded 1. Values shown are Hedge’s *g*. Orange indicates a higher pMD in SCA7 patients versus HVs. Blue indicates a lower pMD in SCA7 patients versus HVs. Results are overlaid on the HV diffusion tensor template.

**Figure S2.4 pFA group comparison effect size map**


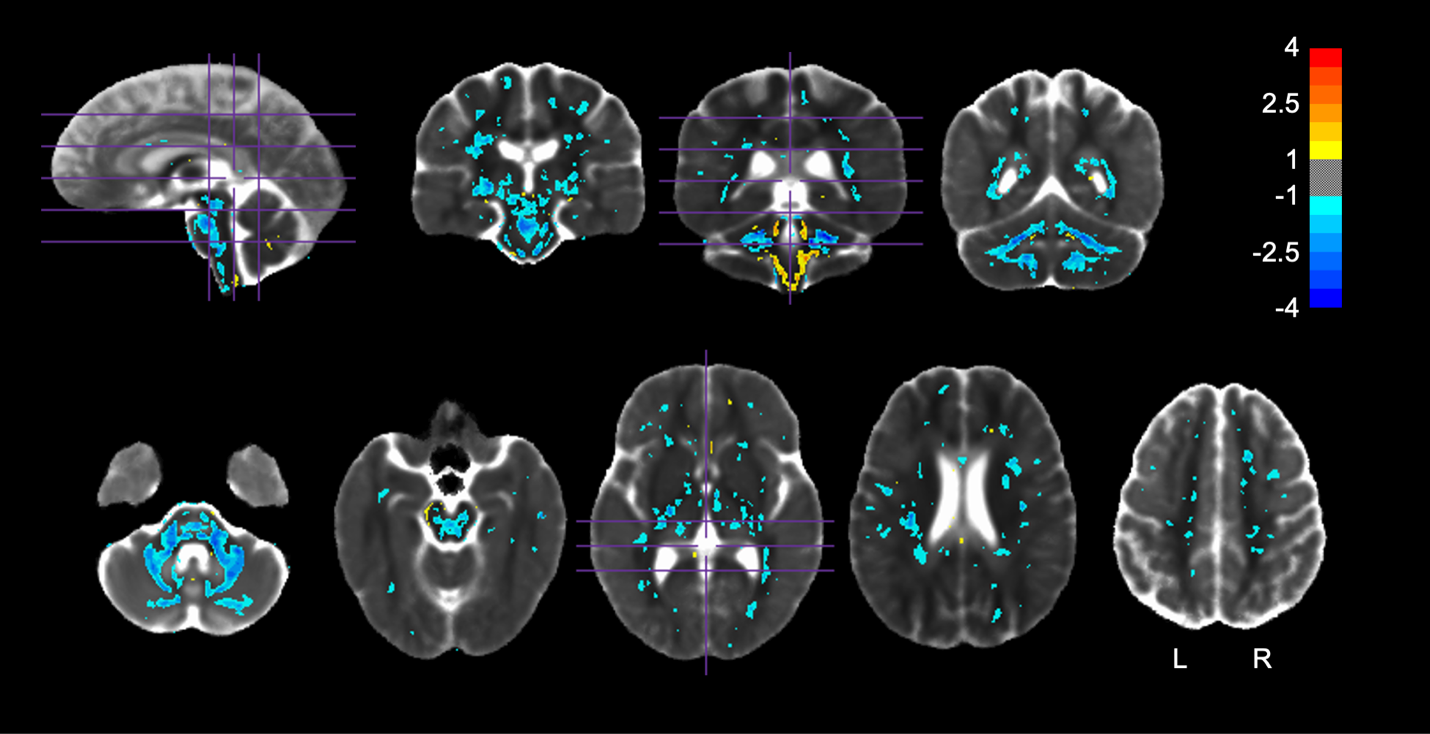


Figure S2.4: Regions in which the absolute value of Hedge’s *g* for the pFA HV versus SCA7 comparison exceeded 1. Values shown are Hedge’s *g*. Blue indicates a lower pFA in SCA7 patients versus HVs. Orange indicates a higher pFA in SCA7 patients versus HVs. Results are overlaid on the HV diffusion tensor template.

**Figure S2.5: VBM group comparison effect size map**


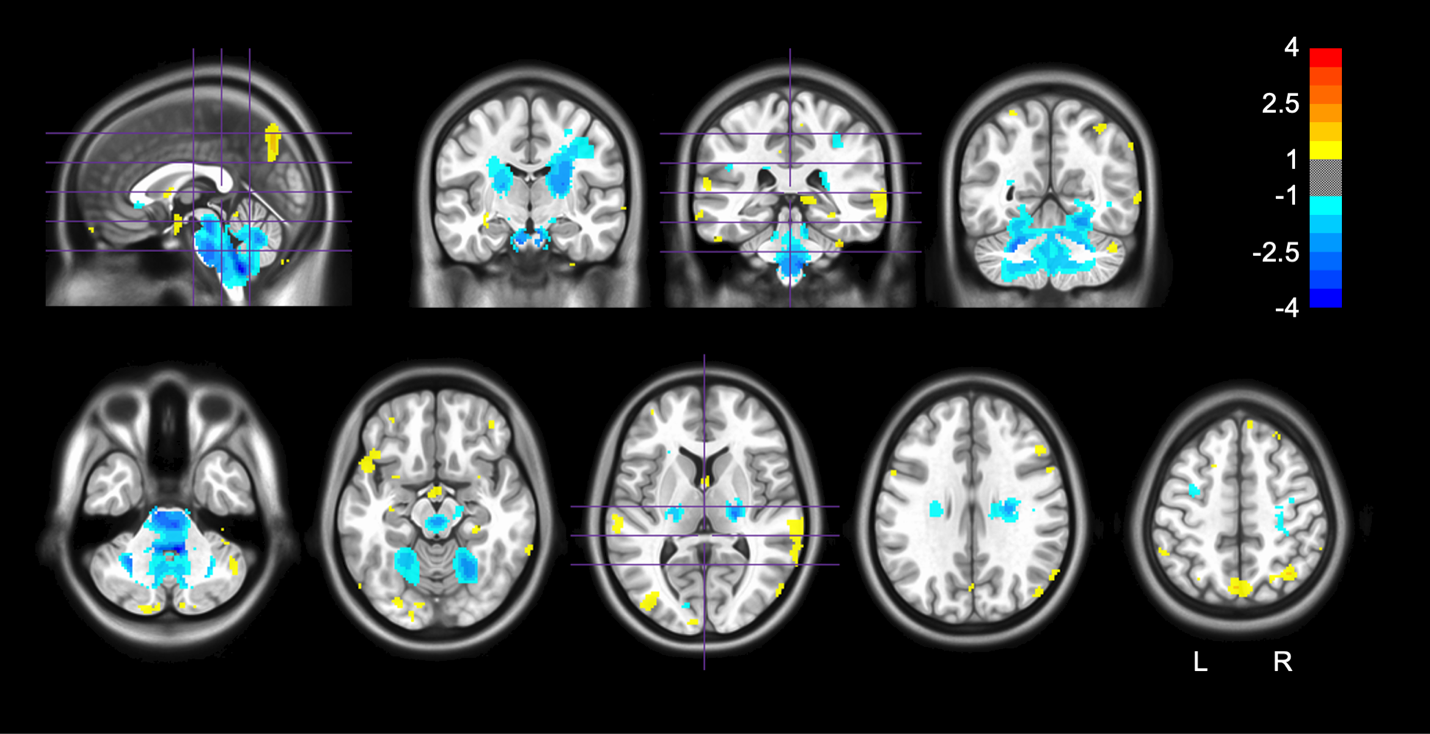


Figure S2.5: Regions in which the absolute value of Hedge’s *g* for the VBM HV versus SCA7 comparison exceeded 1. Values shown are Hedge’s *g*. Blue indicates a lower GM volume in SCA7 patients versus HVs. Orange indicates a higher GM volume. Results are overlaid on the MNI ICBM 2009c Nonlinear Asymmetric template.

**Figure S2.6:** **MD group comparison effect size map**


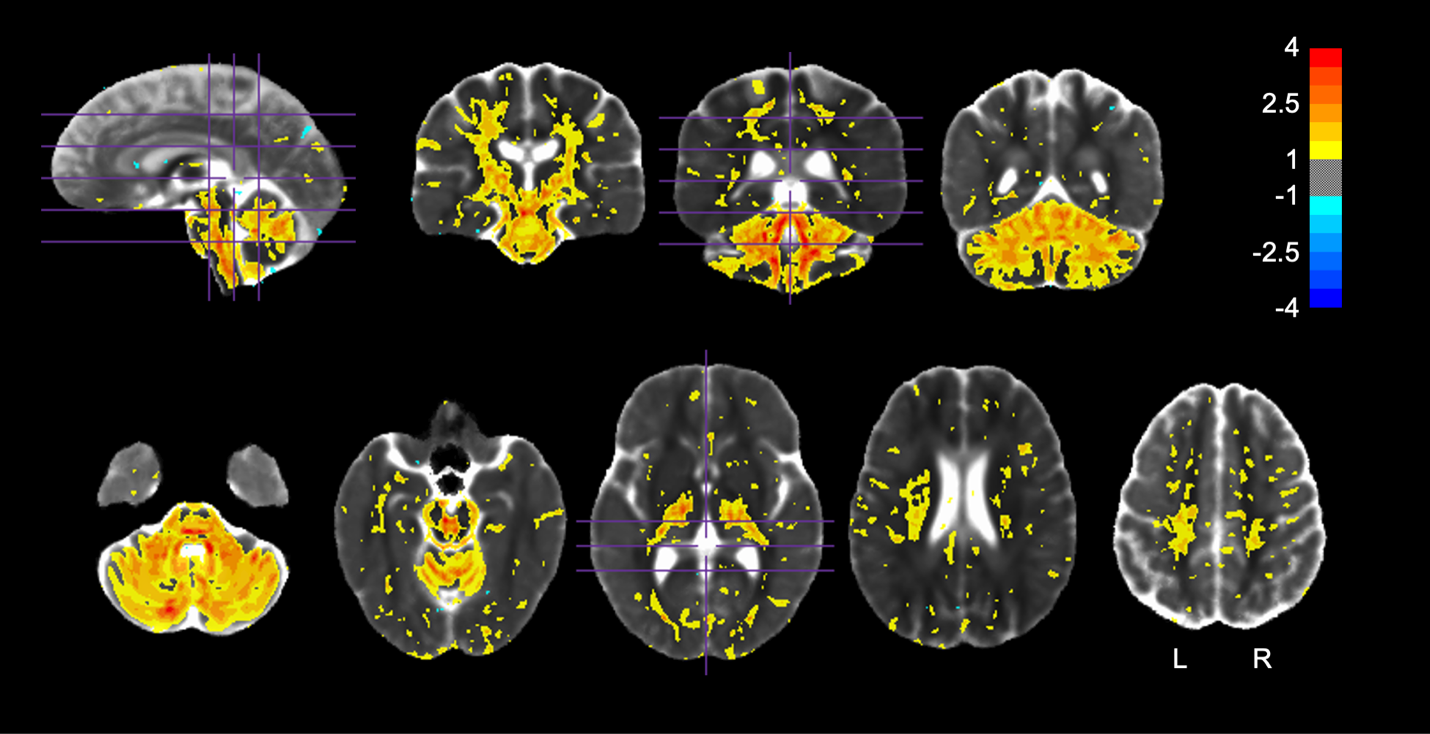


Figure S2.6: Regions in which the absolute value of Hedge’s *g* for the MD HV versus SCA7 comparison exceeded 1. Values shown are Hedge’s *g*. Orange indicates a higher MD in SCA7 patients versus HVs. Blue indicates a lower MD. Results are overlaid on the HV diffusion tensor template.

**Figure S2.7: FA group comparison effect size map**


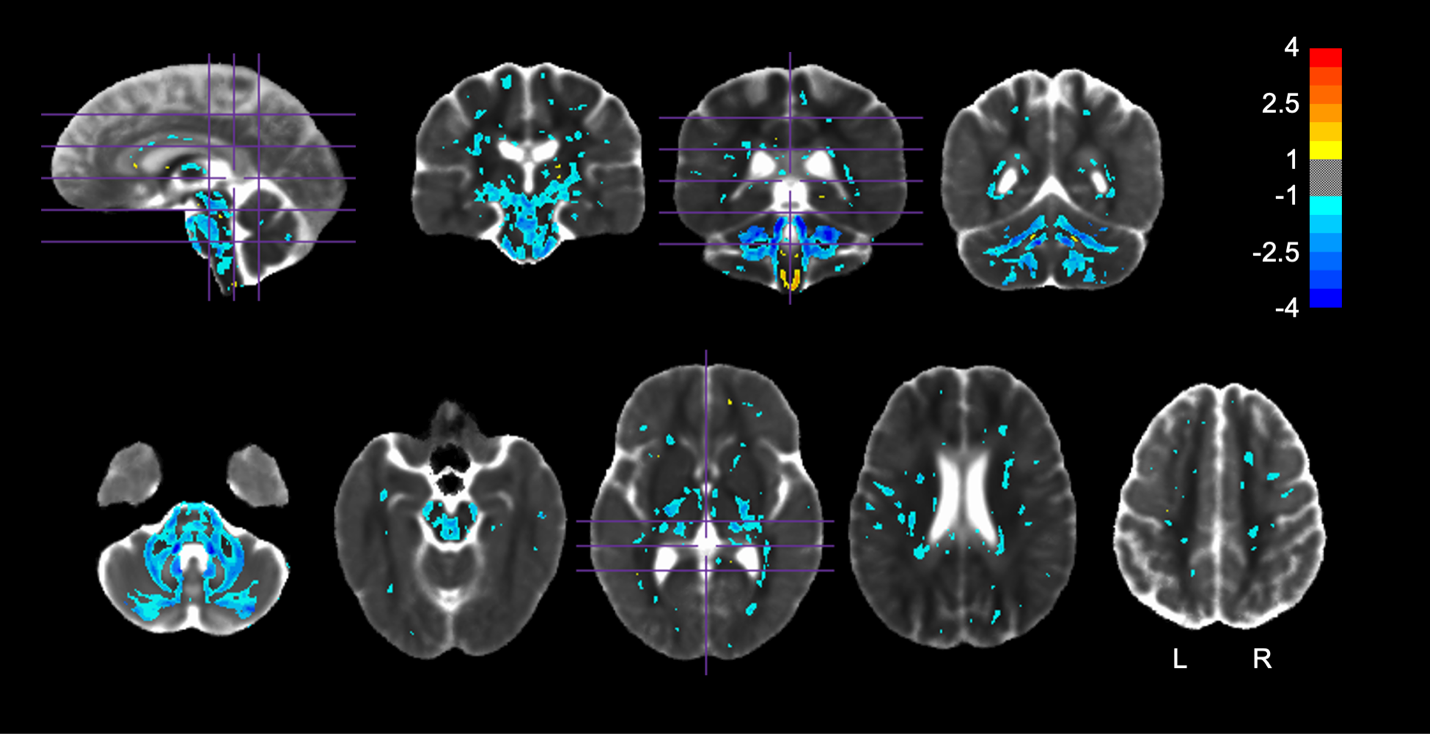


Figure S2.7: Regions in which the absolute value of Hedge’s *g* for the FA HV versus SCA7 comparison exceeded 1. Values shown are Hedge’s *g*. Blue indicates a lower FA in SCA7 patients versus HVs. Orange indicates a higher FA. Results are overlaid on the HV diffusion tensor template.

**S3: VBM, MD, and FA group comparison tables of most affected regions**

**S3.1: VBM tables**

**Table S3.1.1:** VBM FreeSurfer GM atlas

| **ROI** | **Qvoxels** | ***t* peak** | ***g* peak** |
| --- | --- | --- | --- |
| Brain-Stem | 0.794 | -12.066 | -4.570 |
| Left-Cerebellum-Cortex | 0.282 | -11.242 | -4.199 |
| Right-Thalamus-Proper | 0.259 | -7.765 | -3.067 |
| Right-Cerebellum-Cortex | 0.243 | -10.527 | -2.847 |
| Right-VentralDC | 0.234 | -6.782 | -2.270 |
| Left-Thalamus-Proper | 0.199 | -8.238 | -2.122 |
| Left-VentralDC | 0.177 | -6.790 | -2.338 |
| ctx-rh-precentral | 0.107 | -6.153 | -1.760 |

Table S3.1.1: Peak *t­-*statistic value, peak Hedge’s *g* value, and the fraction of ROI voxels significantly different (FWE corrected) between HVs and SCA7 patients (Qvoxels) for the VBM group comparison within ROIs of the FreeSurfer GM atlas. Only regions with Qvoxels ≥ 0.10 are shown.

**S3.2: MD tables**

**Table S3.2.1:** MD ICBM-DTI-81 WM atlas

| **ROI** | **Qvoxels** | ***t* peak** | ***g* peak** |
| --- | --- | --- | --- |
| Pontine crossing tract (a part of MCP) | 0.960 | 11.588 | 4.443 |
| Middle cerebellar peduncle | 0.922 | 11.879 | 4.706 |
| Inferior cerebellar peduncle R | 0.903 | 11.249 | 4.508 |
| Superior cerebellar peduncle R | 0.896 | 14.568 | 5.362 |
| Superior cerebellar peduncle L | 0.838 | 11.237 | 4.472 |
| Inferior cerebellar peduncle L | 0.813 | 10.490 | 4.042 |
| Corticospinal tract L | 0.812 | 7.453 | 2.918 |
| Corticospinal tract R | 0.786 | 7.170 | 2.889 |
| Medial lemniscus L | 0.781 | 10.868 | 3.923 |
| Medial lemniscus R | 0.773 | 11.524 | 4.396 |
| Posterior limb of internal capsule L | 0.772 | 8.300 | 3.254 |
| Superior corona radiata L | 0.735 | 6.240 | 2.372 |
| Fornix (cres) / Stria terminalis R | 0.696 | 5.883 | 2.396 |
| Fornix (cres) / Stria terminalis L | 0.669 | 7.055 | 2.816 |
| Posterior limb of internal capsule R | 0.647 | 6.621 | 2.688 |
| Cerebral peduncle R | 0.538 | 7.082 | 2.862 |
| Cerebral peduncle L | 0.538 | 8.495 | 2.989 |
| Sagittal stratum L | 0.449 | 4.260 | 1.773 |
| Posterior corona radiata L | 0.424 | 4.918 | 1.982 |
| Uncinate fasciculus L | 0.378 | 5.660 | 2.171 |
| Retrolenticular part of internal capsule R | 0.369 | 5.996 | 2.429 |
| Superior corona radiata R | 0.356 | 6.012 | 2.440 |
| Anterior limb of internal capsule L | 0.342 | 6.849 | 2.725 |
| Superior longitudinal fasciculus L | 0.331 | 4.862 | 1.990 |
| Retrolenticular part of internal capsule L | 0.330 | 6.082 | 2.457 |
| Cingulum (cingulate gyrus) L | 0.299 | 5.324 | 1.946 |
| Posterior thalamic radiation L | 0.290 | 4.808 | 1.854 |
| Posterior corona radiata R | 0.280 | 4.441 | 1.833 |
| Superior fronto-occipital fasciculus L | 0.268 | 3.707 | 1.509 |
| Sagittal stratum R | 0.239 | 3.957 | 1.587 |
| Posterior thalamic radiation R | 0.211 | 4.221 | 1.759 |
| Cingulum (hippocampus) R | 0.199 | 4.182 | 1.697 |
| Body of corpus callosum | 0.196 | 4.942 | 1.703 |
| External capsule L | 0.181 | 5.381 | 2.086 |
| Cingulum (cingulate gyrus) R | 0.148 | 4.087 | 1.627 |
| Superior longitudinal fasciculus R | 0.115 | 3.995 | 1.620 |
| Anterior corona radiata L | 0.110 | 4.343 | 1.607 |
| Superior fronto-occipital fasciculus R | 0.110 | 3.528 | 1.497 |

Table S3.2.1: Peak *t­-*statistic value, peak Hedge’s *g* value, and the fraction of ROI voxels significantly different (FWE corrected) between HVs and SCA7 patients (Qvoxels) for the MD group comparison within ROIs of the ICBM-DTI-81 WM atlas. Only regions with Qvoxels ≥ 0.10 are shown.

**Table S3.2.2:** MD FreeSurfer GM atlas

| **ROI** | **Qvoxels** | ***t* peak** | ***g* peak** |
| --- | --- | --- | --- |
| Right-Cerebellum-Cortex | 0.815 | 11.628 | 4.418 |
| Left-Cerebellum-Cortex | 0.807 | 11.504 | 4.558 |
| Brain-Stem | 0.742 | 14.568 | 5.362 |
| Right-Thalamus-Proper | 0.463 | 9.102 | 3.659 |
| Left-Thalamus-Proper | 0.454 | 8.300 | 3.254 |
| Left-VentralDC | 0.442 | 7.482 | 2.965 |
| Right-VentralDC | 0.417 | 7.944 | 3.033 |
| ctx-lh-pericalcarine | 0.358 | 4.846 | 1.801 |
| ctx-rh-pericalcarine | 0.347 | 6.305 | 2.230 |
| ctx-lh-caudalanteriorcingulate | 0.262 | 4.777 | 1.654 |
| ctx-rh-lateraloccipital | 0.233 | 5.761 | 2.188 |
| ctx-rh-posteriorcingulate | 0.228 | 5.867 | 1.645 |
| ctx-rh-isthmuscingulate | 0.226 | 4.400 | 1.595 |
| ctx-rh-paracentral | 0.217 | 7.162 | 2.837 |
| ctx-lh-entorhinal | 0.210 | 4.981 | 1.967 |
| ctx-rh-cuneus | 0.197 | 5.312 | 2.094 |
| ctx-lh-posteriorcingulate | 0.194 | 4.942 | 1.611 |
| Left-Hippocampus | 0.192 | 5.023 | 1.876 |
| ctx-rh-parahippocampal | 0.185 | 5.229 | 2.113 |
| ctx-rh-caudalanteriorcingulate | 0.182 | 4.283 | 1.631 |
| ctx-rh-fusiform | 0.163 | 7.262 | 2.575 |
| ctx-lh-cuneus | 0.161 | 5.405 | 2.072 |
| ctx-lh-lingual | 0.159 | 5.089 | 2.079 |
| Right-Hippocampus | 0.154 | 4.651 | 1.847 |
| ctx-lh-insula | 0.147 | 5.865 | 2.171 |
| ctx-lh-lateraloccipital | 0.139 | 5.871 | 2.003 |
| ctx-rh-lingual | 0.137 | 6.828 | 1.905 |
| ctx-lh-isthmuscingulate | 0.134 | 4.514 | 1.517 |
| ctx-lh-fusiform | 0.129 | 5.619 | 2.188 |
| Left-Putamen | 0.127 | 5.534 | 2.100 |
| ctx-lh-precuneus | 0.123 | 6.319 | 1.701 |
| Right-Pallidum | 0.123 | 4.457 | 1.778 |
| ctx-lh-postcentral | 0.119 | 5.091 | 1.735 |
| ctx-lh-paracentral | 0.117 | 4.604 | 1.594 |
| Left-Amygdala | 0.115 | 4.554 | 1.641 |
| Left-Pallidum | 0.103 | 4.688 | 1.939 |

Table S3.2.2: Peak *t­-*statistic value, peak Hedge’s *g* value, and the fraction of ROI voxels significantly different (FWE corrected) between HVs and SCA7 patients (Qvoxels) for the MD group comparison within ROIs of the FreeSurfer GM atlas. Only regions with Qvoxels ≥ 0.10 are shown.

**S3.3: FA tables**

**Table S3.3.1:** FA ICBM-DTI-81 WM atlas

| **ROI** | **Qvoxels** | ***t* peak** | ***g* peak** |
| --- | --- | --- | --- |
| Superior cerebellar peduncle R | 0.929 | -16.047 | -5.455 |
| Pontine crossing tract (a part of MCP) | 0.910 | -7.921 | -2.995 |
| Superior cerebellar peduncle L | 0.892 | -16.335 | -5.591 |
| Medial lemniscus R | 0.884 | -10.257 | -3.596 |
| Medial lemniscus L | 0.819 | -10.361 | -3.718 |
| Middle cerebellar peduncle | 0.740 | -14.007 | -4.536 |
| Corticospinal tract R | 0.675 | -6.023 | -2.445 |
| Inferior cerebellar peduncle R | 0.671 | -13.507 | -4.956 |
| Corticospinal tract L | 0.626 | -7.951 | -2.969 |
| Cerebral peduncle L | 0.603 | -8.831 | -3.276 |
| Fornix (cres) / Stria terminalis L | 0.574 | -7.380 | -2.972 |
| Fornix (cres) / Stria terminalis R | 0.553 | -7.278 | -2.946 |
| Inferior cerebellar peduncle L | 0.515 | -12.553 | -5.002 |
| Cerebral peduncle R | 0.456 | -7.620 | -2.961 |
| Anterior limb of internal capsule L | 0.443 | -6.877 | -2.755 |
| Posterior thalamic radiation R | 0.432 | -4.702 | -1.945 |
| Retrolenticular part of internal capsule R | 0.411 | -5.508 | -1.831 |
| Posterior thalamic radiation L | 0.378 | -5.519 | -2.250 |
| Posterior limb of internal capsule L | 0.297 | -6.649 | -2.342 |
| Tapetum R | 0.288 | -3.923 | -1.621 |
| Tapetum L | 0.282 | -4.273 | -1.713 |
| Superior corona radiata L | 0.282 | -5.299 | -2.115 |
| Sagittal stratum L | 0.276 | -4.704 | -1.945 |
| Posterior limb of internal capsule R | 0.268 | -6.265 | -2.436 |
| Retrolenticular part of internal capsule L | 0.196 | -5.227 | -1.914 |
| Sagittal stratum R | 0.193 | -4.356 | -1.790 |
| Splenium of corpus callosum | 0.163 | -4.652 | -1.872 |
| Body of corpus callosum | 0.145 | -4.918 | -1.702 |
| Anterior corona radiata L | 0.134 | -5.115 | -1.628 |
| Superior corona radiata R | 0.103 | -4.913 | -1.915 |

Table S3.3.1: Peak *t­-*statistic value, peak Hedge’s *g* value, and the fraction of ROI voxels significantly different (FWE corrected) between HVs and SCA7 patients (Qvoxels) for the FA group comparison within ROIs of the ICBM-DTI-81 WM atlas. Only regions with Qvoxels ≥ 0.10 are shown.

**Table S3.3.2:** FA FreeSurfer GM atlas

| **ROI** | **Qvoxels** | ***t* peak** | ***g* peak** |
| --- | --- | --- | --- |
| Left-Cerebellum-Cortex | 0.829 | -15.155 | -5.471 |
| Right-Cerebellum-Cortex | 0.824 | -15.866 | -5.266 |
| Brain-Stem | 0.639 | -16.335 | -5.591 |
| Left-VentralDC | 0.524 | -8.732 | -3.276 |
| Right-VentralDC | 0.435 | -7.614 | -2.925 |
| Left-Hippocampus | 0.350 | -7.866 | -3.114 |
| Right-Hippocampus | 0.287 | -5.964 | -2.356 |
| Left-Thalamus-Proper | 0.276 | -6.382 | -2.560 |
| Right-Thalamus-Proper | 0.261 | -6.969 | -2.816 |
| Left-Pallidum | 0.184 | -5.069 | -1.991 |
| Right-Pallidum | 0.180 | -6.381 | -2.464 |
| Left-Caudate | 0.136 | -4.602 | -1.833 |
| Left-Putamen | 0.127 | -4.722 | -1.881 |
| Left-Amygdala | 0.118 | -6.477 | -2.105 |

Table S3.3.2: Peak *t­-*statistic value, peak Hedge’s *g* value, and the fraction of ROI voxels significantly different (FWE corrected) between HVs and SCA7 patients (Qvoxels) for the FA group comparison within ROIs of the FreeSurfer GM atlas. Only regions with Qvoxels ≥ 0.10 are shown.

**S4: Correlation analysis supplementary material**

**Figure S4.1: Correlations between imaging metrics and SARA score**

**
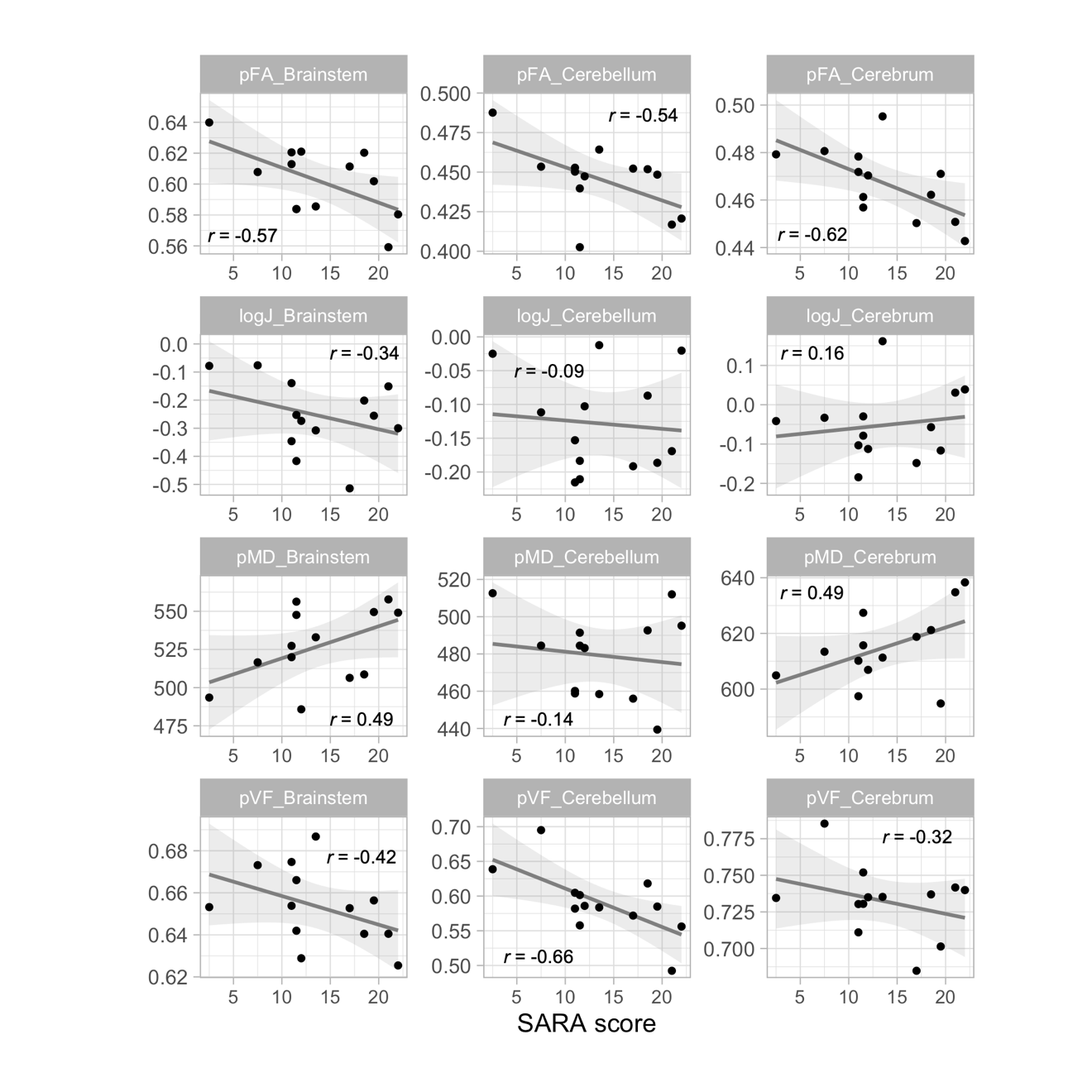
**

Figure S4.1: Scatter plots of the average value of every metric versus the SARA score in the brainstem, cerebellum, and cerebrum. *r* values shown are Pearson’s correlation coefficient. Line and shaded area represent line of best fit and 95% confidence interval respectively.

**Figure S4.2: Correlations of single compartment diffusivity metrics (MD and FA) and dual compartment diffusivity metrics (pMD and pFA) with the parenchymal VF**

**
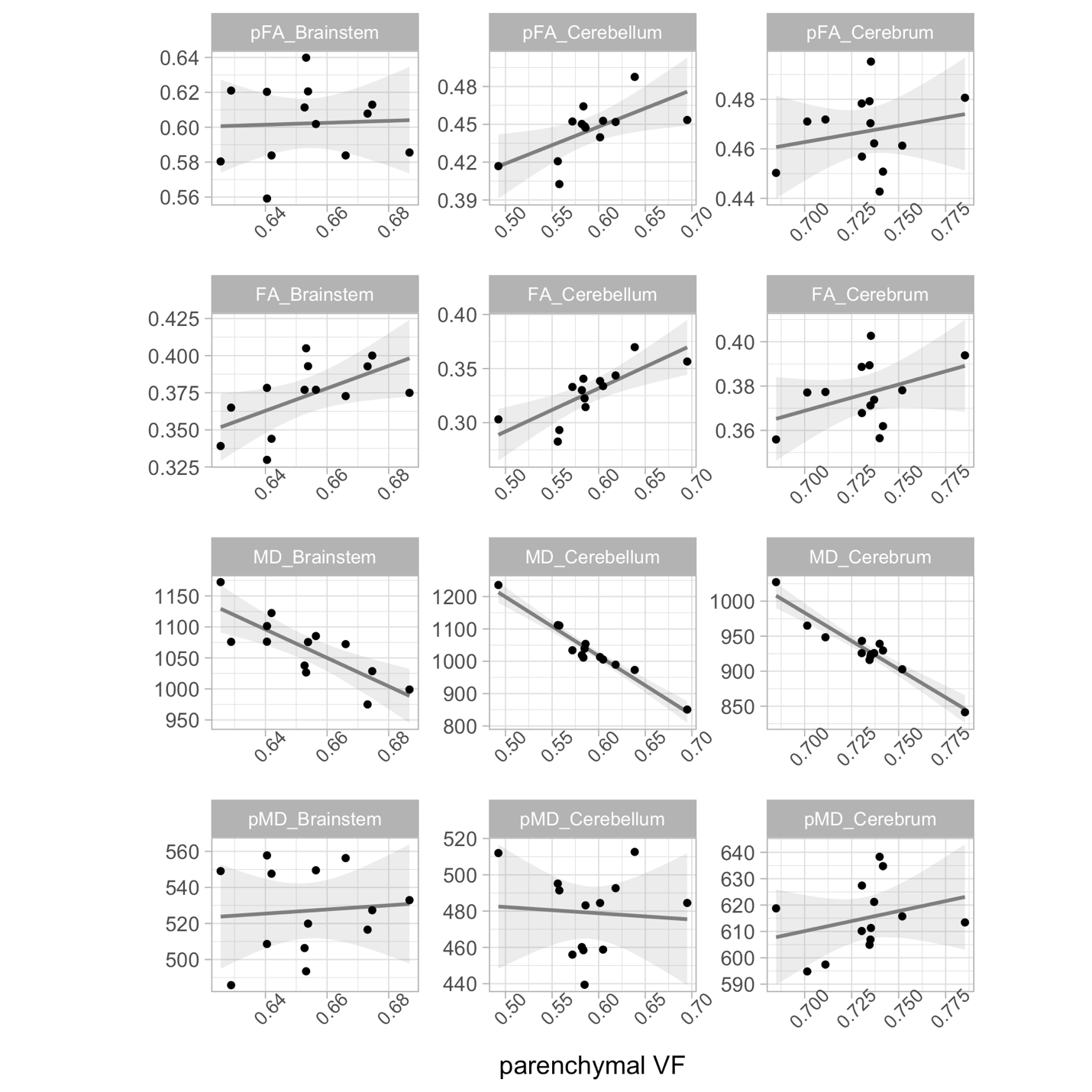
**

Figure S4.2: Scatter plots of the average value of single and dual compartment diffusivity metrics versus the parenchymal VF in the brainstem, cerebellum, and cerebrum. As labeled, pMD = parenchymal (dual compartment) MD, pFA = parenchymal (dual compartment) FA, MD = single compartment MD, and FA = single compartment FA. Line and shaded area represent line of best fit and 95% confidence interval respectively. Compared to the dual compartment diffusivity metrics, the single compartment diffusivity metrics are more strongly correlated with the parenchymal VF. This demonstrates that abnormalities in the single compartment MD and FA may strongly reflect differences in parenchymal tissue volume rather than purely tissue microstructural abnormalities. Given that we observe significant changes in parenchymal VF around in brainstem and cerebellum in patients, we believe the dual-compartment diffusivity metrics are more appropriate for assessing tissue microstructure.

**Figure S4.3: Correlations of whole brain pFA and cerebellar pVF with the SARA score with asymptomatic patient included**


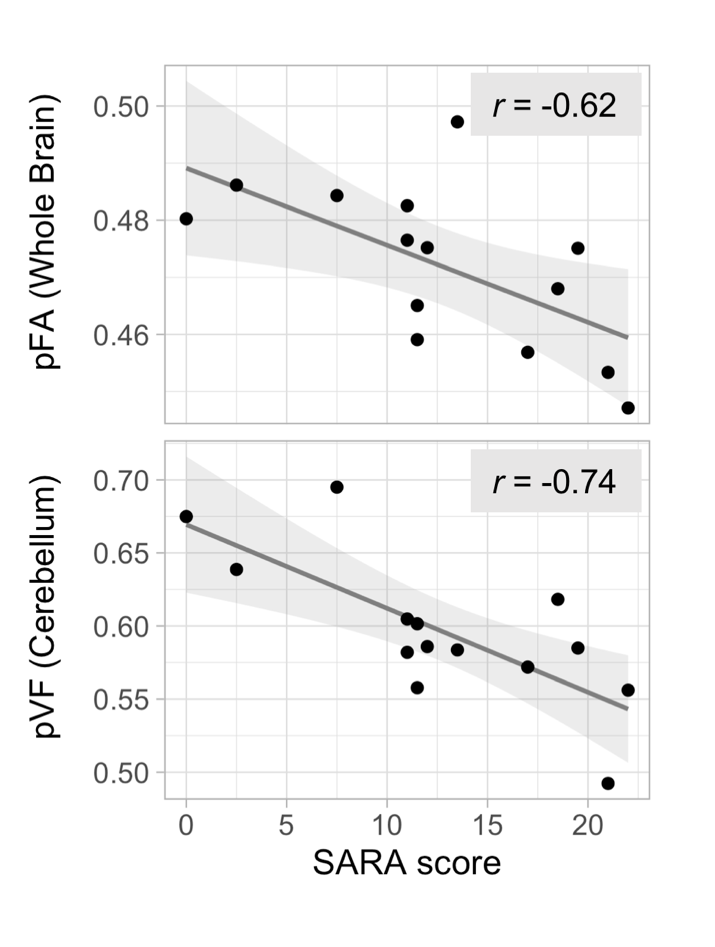


Figure S4.3: Scatter plots of whole brain pFA versus the SARA score (*r* = -0.62, *p* = 0.076, FDR corrected) and cerebellar pVF versus the SARA score (*r* = -0.74, *p* = 0.032, FDR corrected). Line and shaded area represent line of best fit and 95% confidence interval respectively.

**Figure S4.4: Correlations between imaging metrics and SARA score with asymptomatic patient included**


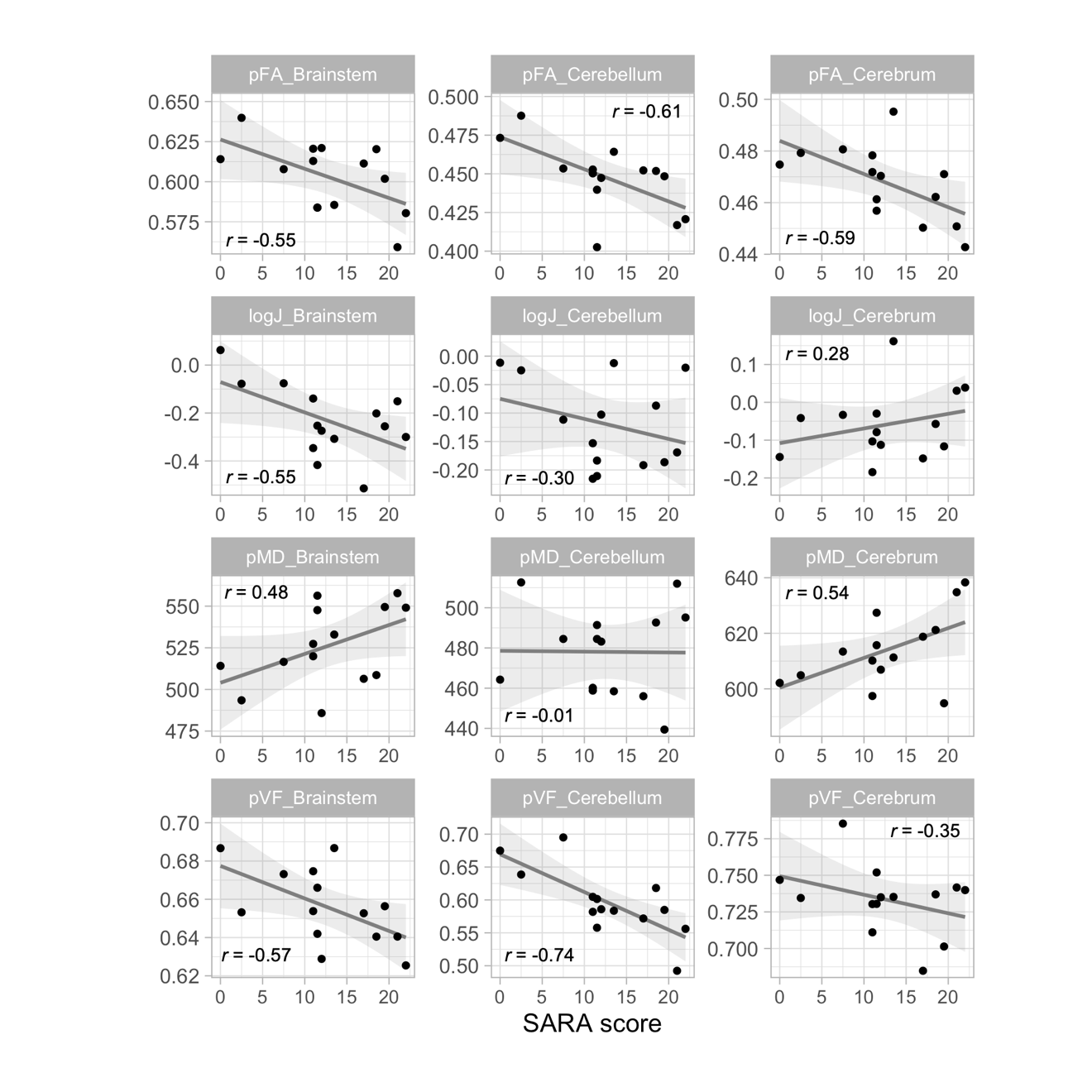


Figure S4.4: Scatter plots of the average value of every metric versus the SARA score in the brainstem, cerebellum, and cerebrum. *r* values shown are Pearson’s correlation coefficient. Line and shaded area represent line of best fit and 95% confidence interval respectively.
