# Supplementary material for: In vivo assessment of neurodegeneration in Spinocerebellar Ataxia type 7
Source: Neuroimage Clin. 2021 Jan 19;29:102561. doi: 10.1016/j.nicl.2021.102561 (PMC7848632; doi:10.1016/j.nicl.2021.102561)
Supplement: Supplementary data 2 [file mmc2.docx]

**Supplementary Materials 2 of 2**

**Contents**

**S5: Group comparison all metrics full atlas tables**

**Table S5.1:** All metrics full ICBM-DTI-81 WM atlas

**Table S5.2:** All metrics full FreeSurfer GM atlas

**S6: Group comparison full atlas tables**

**S6.1:** DTBM (logJ) full tables

**Table S6.1.1:** DTBM (logJ) full ICBM-DTI-81 WM atlas

**Table S6.1.2:** DTBM (logJ) full FreeSurfer GM atlas

**S6.2:** pVF full tables

**Table S6.2.1:** pVF full ICBM-DTI-81 WM atlas

**Table S6.2.2:** pVF full FreeSurfer GM atlas

**S6.3:** pMD full tables

**Table S6.3.1:** pMD full ICBM-DTI-81 WM atlas

**Table S6.3.2:** pMD full FreeSurfer GM atlas

**S6.4:** pFA full tables

**Table S6.4.1:** pFA full ICBM-DTI-81 WM atlas

**Table S6.4.2:** pFA full FreeSurfer GM atlas

**S6.5:** VBM full table

**Table S6.5.1:** VBM full FreeSurfer GM atlas

**S6.6:** MD full tables

**Table S6.6.1:** MD full ICBM-DTI-81 WM atlas

**Table S6.6.2:** MD full FreeSurfer GM atlas

**S6.7:** FA full tables

**Table S6.7.1:** FA full ICBM-DTI-81 WM atlas

**Table S6.7.2:** FA full FreeSurfer GM atlas

**S5: Group comparison all metrics full atlas tables**

**Table S5.1:** All metrics full ICBM-DTI-81 WM atlas table

| **ROI** | **DTBM** | **pVF** | **pMD** | **MD** | **pFA** | **FA** |
| --- | --- | --- | --- | --- | --- | --- |
| Middle cerebellar peduncle | 0.826 | 0.304 | 0.608 | 0.922 | 0.550 | 0.740 |
| Pontine crossing tract (a part of MCP) | 0.997 | 0.000 | 0.812 | 0.960 | 0.806 | 0.910 |
| Genu of corpus callosum | 0.000 | 0.000 | 0.123 | 0.074 | 0.005 | 0.058 |
| Body of corpus callosum | 0.040 | 0.008 | 0.192 | 0.196 | 0.124 | 0.145 |
| Splenium of corpus callosum | 0.019 | 0.010 | 0.137 | 0.079 | 0.203 | 0.163 |
| Fornix (column and body of fornix) | 0.004 | 0.000 | 0.000 | 0.023 | 0.000 | 0.010 |
| Corticospinal tract R | 0.884 | 0.063 | 0.619 | 0.786 | 0.579 | 0.675 |
| Corticospinal tract L | 0.911 | 0.035 | 0.641 | 0.812 | 0.510 | 0.626 |
| Medial lemniscus R | 0.974 | 0.300 | 0.423 | 0.773 | 0.525 | 0.884 |
| Medial lemniscus L | 0.895 | 0.277 | 0.328 | 0.781 | 0.438 | 0.819 |
| Inferior cerebellar peduncle R | 0.843 | 0.573 | 0.297 | 0.903 | 0.311 | 0.671 |
| Inferior cerebellar peduncle L | 0.822 | 0.439 | 0.313 | 0.813 | 0.203 | 0.515 |
| Superior cerebellar peduncle R | 0.548 | 0.705 | 0.162 | 0.896 | 0.143 | 0.929 |
| Superior cerebellar peduncle L | 0.514 | 0.635 | 0.222 | 0.838 | 0.242 | 0.892 |
| Cerebral peduncle R | 0.606 | 0.141 | 0.321 | 0.538 | 0.349 | 0.456 |
| Cerebral peduncle L | 0.679 | 0.175 | 0.312 | 0.538 | 0.413 | 0.603 |
| Anterior limb of internal capsule R | 0.051 | 0.000 | 0.044 | 0.010 | 0.027 | 0.004 |
| Anterior limb of internal capsule L | 0.296 | 0.000 | 0.151 | 0.342 | 0.384 | 0.443 |
| Posterior limb of internal capsule R | 0.449 | 0.000 | 0.318 | 0.647 | 0.266 | 0.268 |
| Posterior limb of internal capsule L | 0.387 | 0.000 | 0.577 | 0.772 | 0.315 | 0.297 |
| Retrolenticular part of internal capsule R | 0.189 | 0.000 | 0.290 | 0.369 | 0.326 | 0.411 |
| Retrolenticular part of internal capsule L | 0.112 | 0.000 | 0.363 | 0.330 | 0.267 | 0.196 |
| Anterior corona radiata R | 0.049 | 0.000 | 0.135 | 0.025 | 0.021 | 0.004 |
| Anterior corona radiata L | 0.062 | 0.000 | 0.181 | 0.110 | 0.121 | 0.134 |
| Superior corona radiata R | 0.504 | 0.000 | 0.505 | 0.356 | 0.279 | 0.103 |
| Superior corona radiata L | 0.426 | 0.000 | 0.550 | 0.735 | 0.346 | 0.282 |
| Posterior corona radiata R | 0.164 | 0.000 | 0.338 | 0.280 | 0.064 | 0.037 |
| Posterior corona radiata L | 0.162 | 0.000 | 0.468 | 0.424 | 0.204 | 0.089 |
| Posterior thalamic radiation R | 0.000 | 0.000 | 0.658 | 0.211 | 0.537 | 0.432 |
| Posterior thalamic radiation L | 0.000 | 0.000 | 0.515 | 0.290 | 0.483 | 0.378 |
| Sagittal stratum R | 0.000 | 0.000 | 0.292 | 0.239 | 0.248 | 0.193 |
| Sagittal stratum L | 0.006 | 0.000 | 0.430 | 0.449 | 0.329 | 0.276 |
| External capsule R | 0.035 | 0.000 | 0.031 | 0.004 | 0.020 | 0.014 |
| External capsule L | 0.010 | 0.000 | 0.131 | 0.181 | 0.129 | 0.087 |
| Cingulum (cingulate gyrus) R | 0.000 | 0.000 | 0.048 | 0.148 | 0.023 | 0.007 |
| Cingulum (cingulate gyrus) L | 0.000 | 0.007 | 0.060 | 0.299 | 0.106 | 0.097 |
| Cingulum (hippocampus) R | 0.000 | 0.021 | 0.005 | 0.199 | 0.013 | 0.030 |
| Cingulum (hippocampus) L | 0.000 | 0.019 | 0.033 | 0.042 | 0.000 | 0.000 |
| Fornix (cres) / Stria terminalis R | 0.446 | 0.131 | 0.300 | 0.696 | 0.212 | 0.553 |
| Fornix (cres) / Stria terminalis L | 0.548 | 0.095 | 0.366 | 0.669 | 0.359 | 0.574 |
| Superior longitudinal fasciculus R | 0.000 | 0.000 | 0.306 | 0.115 | 0.161 | 0.010 |
| Superior longitudinal fasciculus L | 0.026 | 0.000 | 0.302 | 0.331 | 0.091 | 0.038 |
| Superior fronto-occipital fasciculus R | 0.240 | 0.000 | 0.163 | 0.110 | 0.107 | 0.000 |
| Superior fronto-occipital fasciculus L | 0.324 | 0.000 | 0.161 | 0.268 | 0.085 | 0.044 |
| Uncinate fasciculus R | 0.000 | 0.000 | 0.000 | 0.000 | 0.000 | 0.000 |
| Uncinate fasciculus L | 0.000 | 0.000 | 0.112 | 0.378 | 0.000 | 0.000 |
| Tapetum R | 0.157 | 0.000 | 0.236 | 0.090 | 0.249 | 0.288 |
| Tapetum L | 0.082 | 0.001 | 0.057 | 0.020 | 0.182 | 0.282 |


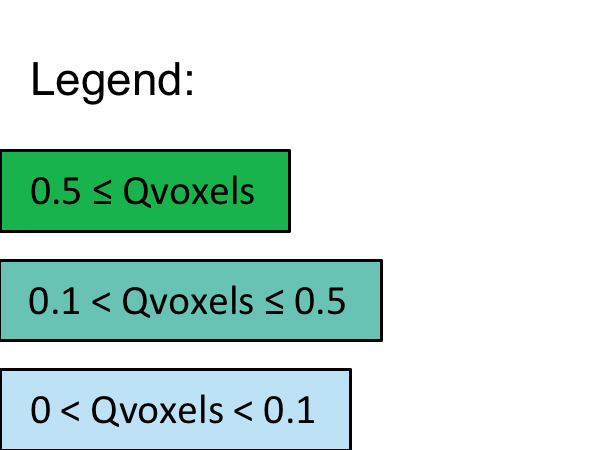


Table S5.1: The fraction of ROI voxels significantly different (FWE corrected) between HVs and SCA7 patients (Qvoxels) for the group comparison of each imaging metric within each ROI of the ICBM-DTI-81 WM atlas.

**Table S5.2:** All metrics FreeSurfer GM atlas table

| **ROI** | **DTBM** | **VBM** | **pVF** | **pMD** | **MD** | **pFA** | **FA** |
| --- | --- | --- | --- | --- | --- | --- | --- |
| Left-Cerebellum-Cortex | 0.411 | 0.282 | 0.611 | 0.098 | 0.807 | 0.548 | 0.829 |
| Left-Thalamus-Proper | 0.550 | 0.199 | 0.016 | 0.256 | 0.454 | 0.168 | 0.276 |
| Left-Caudate | 0.007 | 0.018 | 0.000 | 0.026 | 0.066 | 0.064 | 0.136 |
| Left-Putamen | 0.011 | 0.015 | 0.000 | 0.075 | 0.127 | 0.163 | 0.127 |
| Left-Pallidum | 0.035 | 0.030 | 0.000 | 0.185 | 0.103 | 0.180 | 0.184 |
| Brain-Stem | 0.731 | 0.794 | 0.219 | 0.408 | 0.742 | 0.370 | 0.639 |
| Left-Hippocampus | 0.101 | 0.007 | 0.142 | 0.065 | 0.192 | 0.009 | 0.350 |
| Left-Amygdala | 0.018 | 0.000 | 0.034 | 0.112 | 0.115 | 0.026 | 0.118 |
| Left-Accumbens-area | 0.000 | 0.000 | 0.000 | 0.000 | 0.000 | 0.000 | 0.000 |
| Left-VentralDC | 0.554 | 0.177 | 0.154 | 0.192 | 0.442 | 0.302 | 0.524 |
| Right-Cerebellum-Cortex | 0.412 | 0.243 | 0.588 | 0.151 | 0.815 | 0.608 | 0.824 |
| Right-Thalamus-Proper | 0.565 | 0.259 | 0.025 | 0.215 | 0.463 | 0.176 | 0.261 |
| Right-Caudate | 0.033 | 0.079 | 0.000 | 0.007 | 0.027 | 0.002 | 0.002 |
| Right-Putamen | 0.014 | 0.078 | 0.000 | 0.016 | 0.014 | 0.030 | 0.029 |
| Right-Pallidum | 0.079 | 0.080 | 0.000 | 0.177 | 0.123 | 0.183 | 0.180 |
| Right-Hippocampus | 0.050 | 0.020 | 0.101 | 0.006 | 0.154 | 0.000 | 0.287 |
| Right-Amygdala | 0.000 | 0.000 | 0.019 | 0.002 | 0.004 | 0.000 | 0.051 |
| Right-Accumbens-area | 0.000 | 0.000 | 0.000 | 0.000 | 0.000 | 0.000 | 0.000 |
| Right-VentralDC | 0.445 | 0.234 | 0.145 | 0.162 | 0.417 | 0.186 | 0.435 |
| ctx-lh-bankssts | 0.000 | 0.000 | 0.000 | 0.000 | 0.002 | NA | NA |
| ctx-lh-caudalanteriorcingulate | 0.000 | 0.000 | 0.000 | 0.040 | 0.262 | NA | NA |
| ctx-lh-caudalmiddlefrontal | 0.000 | 0.000 | 0.000 | 0.113 | 0.065 | NA | NA |
| ctx-lh-cuneus | 0.078 | 0.000 | 0.030 | 0.102 | 0.161 | NA | NA |
| ctx-lh-entorhinal | 0.013 | 0.000 | 0.072 | 0.032 | 0.210 | NA | NA |
| ctx-lh-fusiform | 0.000 | 0.054 | 0.031 | 0.050 | 0.129 | NA | NA |
| ctx-lh-inferiorparietal | 0.000 | 0.000 | 0.000 | 0.089 | 0.026 | NA | NA |
| ctx-lh-inferiortemporal | 0.000 | 0.000 | 0.000 | 0.034 | 0.051 | NA | NA |
| ctx-lh-isthmuscingulate | 0.000 | 0.005 | 0.029 | 0.013 | 0.134 | NA | NA |
| ctx-lh-lateraloccipital | 0.001 | 0.000 | 0.015 | 0.094 | 0.139 | NA | NA |
| ctx-lh-lateralorbitofrontal | 0.000 | 0.000 | 0.000 | 0.062 | 0.004 | NA | NA |
| ctx-lh-lingual | 0.108 | 0.089 | 0.003 | 0.091 | 0.159 | NA | NA |
| ctx-lh-medialorbitofrontal | 0.001 | 0.000 | 0.000 | 0.017 | 0.001 | NA | NA |
| ctx-lh-middletemporal | 0.000 | 0.000 | 0.000 | 0.068 | 0.006 | NA | NA |
| ctx-lh-parahippocampal | 0.030 | 0.000 | 0.014 | 0.009 | 0.074 | NA | NA |
| ctx-lh-paracentral | 0.000 | 0.000 | 0.000 | 0.073 | 0.117 | NA | NA |
| ctx-lh-parsopercularis | 0.000 | 0.000 | 0.000 | 0.066 | 0.080 | NA | NA |
| ctx-lh-parsorbitalis | 0.000 | 0.000 | 0.000 | 0.032 | 0.000 | NA | NA |
| ctx-lh-parstriangularis | 0.000 | 0.000 | 0.000 | 0.003 | 0.003 | NA | NA |
| ctx-lh-pericalcarine | 0.028 | 0.000 | 0.020 | 0.029 | 0.358 | NA | NA |
| ctx-lh-postcentral | 0.000 | 0.001 | 0.000 | 0.136 | 0.119 | NA | NA |
| ctx-lh-posteriorcingulate | 0.002 | 0.000 | 0.020 | 0.028 | 0.194 | NA | NA |
| ctx-lh-precentral | 0.000 | 0.021 | 0.000 | 0.139 | 0.089 | NA | NA |
| ctx-lh-precuneus | 0.051 | 0.000 | 0.003 | 0.109 | 0.123 | NA | NA |
| ctx-lh-rostralanteriorcingulate | 0.000 | 0.000 | 0.000 | 0.031 | 0.051 | NA | NA |
| ctx-lh-rostralmiddlefrontal | 0.000 | 0.000 | 0.000 | 0.104 | 0.039 | NA | NA |
| ctx-lh-superiorfrontal | 0.000 | 0.000 | 0.000 | 0.160 | 0.063 | NA | NA |
| ctx-lh-superiorparietal | 0.000 | 0.001 | 0.000 | 0.131 | 0.075 | NA | NA |
| ctx-lh-superiortemporal | 0.000 | 0.000 | 0.000 | 0.035 | 0.014 | NA | NA |
| ctx-lh-supramarginal | 0.000 | 0.000 | 0.000 | 0.063 | 0.027 | NA | NA |
| ctx-lh-frontalpole | 0.000 | 0.000 | 0.000 | 0.074 | 0.000 | NA | NA |
| ctx-lh-temporalpole | 0.000 | 0.000 | 0.027 | 0.002 | 0.034 | NA | NA |
| ctx-lh-transversetemporal | 0.000 | 0.000 | 0.000 | 0.039 | 0.011 | NA | NA |
| ctx-lh-insula | 0.002 | 0.000 | 0.000 | 0.030 | 0.147 | NA | NA |
| ctx-rh-bankssts | 0.000 | 0.000 | 0.000 | 0.026 | 0.016 | NA | NA |
| ctx-rh-caudalanteriorcingulate | 0.000 | 0.000 | 0.000 | 0.035 | 0.182 | NA | NA |
| ctx-rh-caudalmiddlefrontal | 0.000 | 0.000 | 0.000 | 0.085 | 0.014 | NA | NA |
| ctx-rh-cuneus | 0.025 | 0.000 | 0.011 | 0.078 | 0.197 | NA | NA |
| ctx-rh-entorhinal | 0.007 | 0.003 | 0.071 | 0.000 | 0.065 | NA | NA |
| ctx-rh-fusiform | 0.013 | 0.069 | 0.061 | 0.086 | 0.163 | NA | NA |
| ctx-rh-inferiorparietal | 0.000 | 0.000 | 0.000 | 0.092 | 0.083 | NA | NA |
| ctx-rh-inferiortemporal | 0.000 | 0.000 | 0.000 | 0.017 | 0.024 | NA | NA |
| ctx-rh-isthmuscingulate | 0.000 | 0.000 | 0.073 | 0.018 | 0.226 | NA | NA |
| ctx-rh-lateraloccipital | 0.000 | 0.000 | 0.021 | 0.124 | 0.233 | NA | NA |
| ctx-rh-lateralorbitofrontal | 0.001 | 0.000 | 0.000 | 0.000 | 0.000 | NA | NA |
| ctx-rh-lingual | 0.093 | 0.036 | 0.016 | 0.103 | 0.137 | NA | NA |
| ctx-rh-medialorbitofrontal | 0.000 | 0.000 | 0.000 | 0.000 | 0.000 | NA | NA |
| ctx-rh-middletemporal | 0.000 | 0.000 | 0.000 | 0.004 | 0.037 | NA | NA |
| ctx-rh-parahippocampal | 0.030 | 0.000 | 0.049 | 0.001 | 0.185 | NA | NA |
| ctx-rh-paracentral | 0.000 | 0.000 | 0.000 | 0.158 | 0.217 | NA | NA |
| ctx-rh-parsopercularis | 0.000 | 0.000 | 0.000 | 0.029 | 0.002 | NA | NA |
| ctx-rh-parsorbitalis | 0.000 | 0.000 | 0.000 | 0.000 | 0.000 | NA | NA |
| ctx-rh-parstriangularis | 0.000 | 0.000 | 0.000 | 0.095 | 0.001 | NA | NA |
| ctx-rh-pericalcarine | 0.001 | 0.000 | 0.022 | 0.083 | 0.347 | NA | NA |
| ctx-rh-postcentral | 0.000 | 0.053 | 0.000 | 0.047 | 0.064 | NA | NA |
| ctx-rh-posteriorcingulate | 0.027 | 0.004 | 0.000 | 0.028 | 0.228 | NA | NA |
| ctx-rh-precentral | 0.000 | 0.107 | 0.000 | 0.068 | 0.062 | NA | NA |
| ctx-rh-precuneus | 0.056 | 0.000 | 0.000 | 0.097 | 0.087 | NA | NA |
| ctx-rh-rostralanteriorcingulate | 0.000 | 0.000 | 0.000 | 0.008 | 0.005 | NA | NA |
| ctx-rh-rostralmiddlefrontal | 0.000 | 0.000 | 0.000 | 0.047 | 0.000 | NA | NA |
| ctx-rh-superiorfrontal | 0.000 | 0.000 | 0.000 | 0.056 | 0.057 | NA | NA |
| ctx-rh-superiorparietal | 0.007 | 0.020 | 0.000 | 0.109 | 0.079 | NA | NA |
| ctx-rh-superiortemporal | 0.000 | 0.000 | 0.000 | 0.001 | 0.011 | NA | NA |
| ctx-rh-supramarginal | 0.000 | 0.011 | 0.000 | 0.075 | 0.033 | NA | NA |
| ctx-rh-frontalpole | 0.000 | 0.000 | 0.000 | 0.000 | 0.000 | NA | NA |
| ctx-rh-temporalpole | 0.000 | 0.000 | 0.000 | 0.000 | 0.000 | NA | NA |
| ctx-rh-transversetemporal | 0.000 | 0.000 | 0.000 | 0.013 | 0.064 | NA | NA |
| ctx-rh-insula | 0.007 | 0.000 | 0.000 | 0.001 | 0.021 | NA | NA |


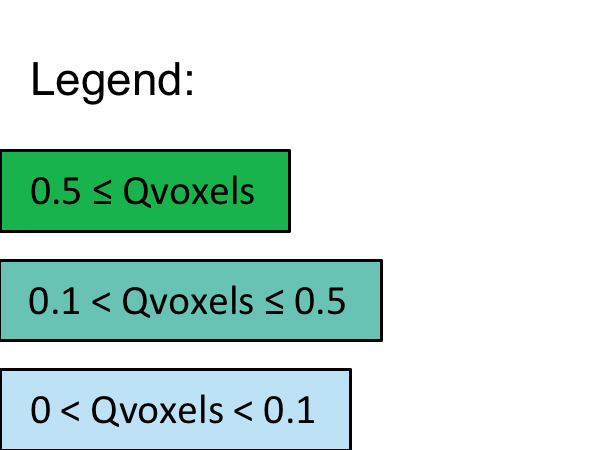


Table S5.2: The fraction of ROI voxels significantly different (FWE corrected) between HVs and SCA7 patients (Qvoxels) for the group comparison of each imaging metric within each ROI of the FreeSurfer GM atlas.

**S6: Group comparison full atlas tables**

**S6.1:** DTBM (logJ) full tables

**Table S6.1.1:** DTBM (logJ) full ICBM-DTI-81 WM atlas table

| **ROI** | ***t*** peak | ***g*** peak | **Qvoxels** |
| --- | --- | --- | --- |
| Middle cerebellar peduncle | -10.534 | -3.882 | 0.826 |
| Pontine crossing tract (a part of MCP) | -10.455 | -3.583 | 0.997 |
| Genu of corpus callosum | -2.824 | -0.604 | 0.000 |
| Body of corpus callosum | -5.726 | -2.068 | 0.040 |
| Splenium of corpus callosum | -3.813 | -1.304 | 0.019 |
| Fornix (column and body of fornix) | -2.584 | -1.044 | 0.004 |
| Corticospinal tract R | -9.272 | -3.090 | 0.884 |
| Corticospinal tract L | -9.103 | -3.061 | 0.911 |
| Medial lemniscus R | -13.412 | -4.443 | 0.974 |
| Medial lemniscus L | -11.353 | -4.439 | 0.895 |
| Inferior cerebellar peduncle R | -13.367 | -5.412 | 0.843 |
| Inferior cerebellar peduncle L | -12.846 | -5.309 | 0.822 |
| Superior cerebellar peduncle R | -13.362 | -4.735 | 0.548 |
| Superior cerebellar peduncle L | -8.964 | -3.532 | 0.514 |
| Cerebral peduncle R | -10.466 | -2.840 | 0.606 |
| Cerebral peduncle L | -11.885 | -2.682 | 0.679 |
| Anterior limb of internal capsule R | -4.255 | -1.823 | 0.051 |
| Anterior limb of internal capsule L | -6.626 | -2.382 | 0.296 |
| Posterior limb of internal capsule R | -9.659 | -2.100 | 0.449 |
| Posterior limb of internal capsule L | -9.206 | -2.157 | 0.387 |
| Retrolenticular part of internal capsule R | -5.109 | -1.431 | 0.189 |
| Retrolenticular part of internal capsule L | -4.132 | -1.205 | 0.112 |
| Anterior corona radiata R | -5.114 | -1.628 | 0.049 |
| Anterior corona radiata L | -3.940 | -1.482 | 0.062 |
| Superior corona radiata R | -5.668 | -2.420 | 0.504 |
| Superior corona radiata L | -6.781 | -2.658 | 0.426 |
| Posterior corona radiata R | -4.286 | -1.655 | 0.164 |
| Posterior corona radiata L | -4.578 | -1.990 | 0.162 |
| Posterior thalamic radiation R | 0.000 | 0.000 | 0.000 |
| Posterior thalamic radiation L | 0.000 | 0.000 | 0.000 |
| Sagittal stratum R | 0.000 | 0.000 | 0.000 |
| Sagittal stratum L | -3.046 | -1.330 | 0.006 |
| External capsule R | -5.388 | -1.922 | 0.035 |
| External capsule L | -3.297 | -1.277 | 0.010 |
| Cingulum (cingulate gyrus) R | 0.000 | 0.000 | 0.000 |
| Cingulum (cingulate gyrus) L | 0.000 | 0.000 | 0.000 |
| Cingulum (hippocampus) R | 0.000 | 0.000 | 0.000 |
| Cingulum (hippocampus) L | 0.000 | 0.000 | 0.000 |
| Fornix (cres) / Stria terminalis R | -5.680 | -2.361 | 0.446 |
| Fornix (cres) / Stria terminalis L | -7.330 | -2.288 | 0.548 |
| Superior longitudinal fasciculus R | 0.000 | 0.000 | 0.000 |
| Superior longitudinal fasciculus L | -3.339 | -1.143 | 0.026 |
| Superior fronto-occipital fasciculus R | -4.605 | -1.991 | 0.240 |
| Superior fronto-occipital fasciculus L | -5.707 | -2.039 | 0.324 |
| Uncinate fasciculus R | 0.000 | 0.000 | 0.000 |
| Uncinate fasciculus L | 0.000 | 0.000 | 0.000 |
| Tapetum R | -3.356 | -1.500 | 0.157 |
| Tapetum L | -3.609 | -1.605 | 0.082 |


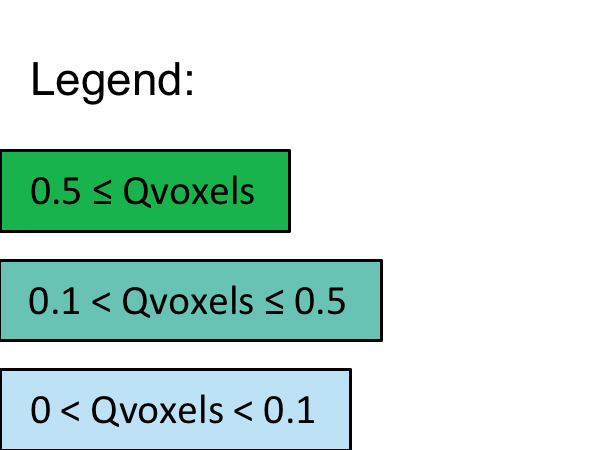


Table S6.1.1: Peak *t­* statistic value, peak Hedge’s *g* value, and the fraction of ROI voxels significantly different (FWE corrected) between HVs and SCA7 patients (Qvoxels) for the DTBM (logJ) group comparison within each ROI of the ICBM-DTI-81 WM atlas.

**Table S6.1.2:** DTBM (logJ) full FreeSurfer GM atlas table

| **ROI** | **t_peak** | **g_peak** | **Qvoxels** |
| --- | --- | --- | --- |
| Left-Cerebellum-Cortex | -9.428 | -3.628 | 0.411 |
| Left-Thalamus-Proper | -8.969 | -2.358 | 0.550 |
| Left-Caudate | -4.296 | -1.585 | 0.007 |
| Left-Putamen | -5.281 | -1.939 | 0.011 |
| Left-Pallidum | -5.877 | -1.660 | 0.035 |
| Brain-Stem | -13.412 | -5.412 | 0.731 |
| Left-Hippocampus | -5.870 | -1.858 | 0.101 |
| Left-Amygdala | -3.917 | -1.318 | 0.018 |
| Left-Accumbens-area | 0.000 | 0.000 | 0.000 |
| Left-VentralDC | -11.885 | -2.834 | 0.554 |
| Right-Cerebellum-Cortex | -10.807 | -4.785 | 0.412 |
| Right-Thalamus-Proper | -8.155 | -2.661 | 0.565 |
| Right-Caudate | -5.393 | -1.841 | 0.033 |
| Right-Putamen | -3.348 | -1.065 | 0.014 |
| Right-Pallidum | -8.617 | -2.100 | 0.079 |
| Right-Hippocampus | -7.364 | -1.959 | 0.050 |
| Right-Amygdala | 0.000 | 0.000 | 0.000 |
| Right-Accumbens-area | 0.000 | 0.000 | 0.000 |
| Right-VentralDC | -8.824 | -2.901 | 0.445 |
| ctx-lh-bankssts | 0.000 | 0.000 | 0.000 |
| ctx-lh-caudalanteriorcingulate | 0.000 | 0.000 | 0.000 |
| ctx-lh-caudalmiddlefrontal | -2.632 | -0.990 | 0.000 |
| ctx-lh-cuneus | 5.493 | 1.398 | 0.078 |
| ctx-lh-entorhinal | -4.968 | -1.884 | 0.013 |
| ctx-lh-fusiform | 6.459 | 1.139 | 0.000 |
| ctx-lh-inferiorparietal | 0.000 | 0.000 | 0.000 |
| ctx-lh-inferiortemporal | 0.000 | 0.000 | 0.000 |
| ctx-lh-isthmuscingulate | 0.000 | 0.000 | 0.000 |
| ctx-lh-lateraloccipital | -3.025 | -1.177 | 0.001 |
| ctx-lh-lateralorbitofrontal | 0.000 | 0.000 | 0.000 |
| ctx-lh-lingual | 6.286 | 1.575 | 0.108 |
| ctx-lh-medialorbitofrontal | -3.175 | -1.394 | 0.001 |
| ctx-lh-middletemporal | 0.000 | 0.000 | 0.000 |
| ctx-lh-parahippocampal | 5.352 | 2.022 | 0.030 |
| ctx-lh-paracentral | 0.000 | 0.000 | 0.000 |
| ctx-lh-parsopercularis | 0.000 | 0.000 | 0.000 |
| ctx-lh-parsorbitalis | 0.000 | 0.000 | 0.000 |
| ctx-lh-parstriangularis | 0.000 | 0.000 | 0.000 |
| ctx-lh-pericalcarine | 4.329 | 0.707 | 0.028 |
| ctx-lh-postcentral | 0.000 | 0.000 | 0.000 |
| ctx-lh-posteriorcingulate | -3.874 | -1.271 | 0.002 |
| ctx-lh-precentral | -3.876 | -1.414 | 0.000 |
| ctx-lh-precuneus | 5.687 | 1.254 | 0.051 |
| ctx-lh-rostralanteriorcingulate | 0.000 | 0.000 | 0.000 |
| ctx-lh-rostralmiddlefrontal | 0.000 | 0.000 | 0.000 |
| ctx-lh-superiorfrontal | -3.400 | -1.255 | 0.000 |
| ctx-lh-superiorparietal | 0.000 | 0.000 | 0.000 |
| ctx-lh-superiortemporal | 0.000 | 0.000 | 0.000 |
| ctx-lh-supramarginal | 0.000 | 0.000 | 0.000 |
| ctx-lh-frontalpole | 0.000 | 0.000 | 0.000 |
| ctx-lh-temporalpole | 0.000 | 0.000 | 0.000 |
| ctx-lh-transversetemporal | 0.000 | 0.000 | 0.000 |
| ctx-lh-insula | -3.390 | -0.960 | 0.002 |
| ctx-rh-bankssts | 0.000 | 0.000 | 0.000 |
| ctx-rh-caudalanteriorcingulate | 0.000 | 0.000 | 0.000 |
| ctx-rh-caudalmiddlefrontal | 0.000 | 0.000 | 0.000 |
| ctx-rh-cuneus | 5.573 | 1.305 | 0.025 |
| ctx-rh-entorhinal | 5.704 | 1.817 | 0.007 |
| ctx-rh-fusiform | 6.081 | 1.582 | 0.013 |
| ctx-rh-inferiorparietal | 0.000 | 0.000 | 0.000 |
| ctx-rh-inferiortemporal | 0.000 | 0.000 | 0.000 |
| ctx-rh-isthmuscingulate | 0.000 | 0.000 | 0.000 |
| ctx-rh-lateraloccipital | 0.000 | 0.000 | 0.000 |
| ctx-rh-lateralorbitofrontal | -3.364 | -0.907 | 0.001 |
| ctx-rh-lingual | 6.531 | 1.630 | 0.093 |
| ctx-rh-medialorbitofrontal | 0.000 | 0.000 | 0.000 |
| ctx-rh-middletemporal | 0.000 | 0.000 | 0.000 |
| ctx-rh-parahippocampal | 4.978 | 2.126 | 0.030 |
| ctx-rh-paracentral | 0.000 | 0.000 | 0.000 |
| ctx-rh-parsopercularis | 0.000 | 0.000 | 0.000 |
| ctx-rh-parsorbitalis | 0.000 | 0.000 | 0.000 |
| ctx-rh-parstriangularis | 0.000 | 0.000 | 0.000 |
| ctx-rh-pericalcarine | 3.449 | 0.552 | 0.001 |
| ctx-rh-postcentral | 0.000 | 0.000 | 0.000 |
| ctx-rh-posteriorcingulate | -3.495 | -1.550 | 0.027 |
| ctx-rh-precentral | 0.000 | 0.000 | 0.000 |
| ctx-rh-precuneus | 6.719 | 1.354 | 0.056 |
| ctx-rh-rostralanteriorcingulate | 0.000 | 0.000 | 0.000 |
| ctx-rh-rostralmiddlefrontal | 0.000 | 0.000 | 0.000 |
| ctx-rh-superiorfrontal | 0.000 | 0.000 | 0.000 |
| ctx-rh-superiorparietal | 5.254 | 1.433 | 0.007 |
| ctx-rh-superiortemporal | 0.000 | 0.000 | 0.000 |
| ctx-rh-supramarginal | 0.000 | 0.000 | 0.000 |
| ctx-rh-frontalpole | 0.000 | 0.000 | 0.000 |
| ctx-rh-temporalpole | 0.000 | 0.000 | 0.000 |
| ctx-rh-transversetemporal | 0.000 | 0.000 | 0.000 |
| ctx-rh-insula | -2.975 | -0.500 | 0.007 |


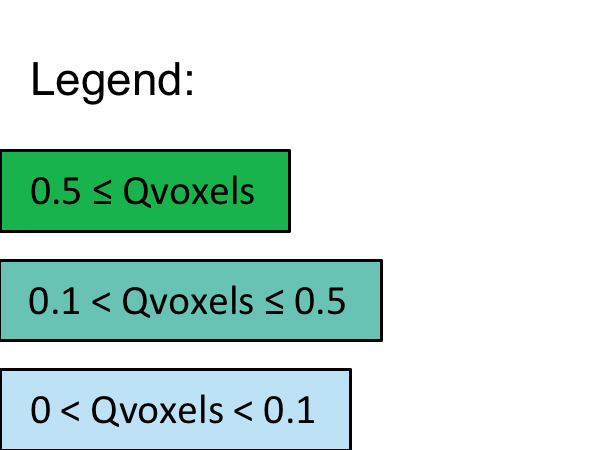


Table S6.1.2: Peak *t­* statistic value, peak Hedge’s *g* value, and the fraction of ROI voxels significantly different (FWE corrected) between HVs and SCA7 patients (Qvoxels) for the DTBM (logJ) group comparison within each ROI of the FreeSurfer GM atlas.

**S6.2:** pVF full tables

**Table S6.2.1:** pVF full ICBM-DTI-81 WM atlas table

| **ROI** | ***t*** peak | ***g*** peak | **Qvoxels** |
| --- | --- | --- | --- |
| Middle cerebellar peduncle | -11.173 | -4.468 | 0.304 |
| Pontine crossing tract (a part of MCP) | 0.000 | 0.000 | 0.000 |
| Genu of corpus callosum | 0.000 | 0.000 | 0.000 |
| Body of corpus callosum | -3.718 | -1.368 | 0.008 |
| Splenium of corpus callosum | -3.618 | -1.278 | 0.010 |
| Fornix (column and body of fornix) | 0.000 | 0.000 | 0.000 |
| Corticospinal tract R | -5.102 | -1.705 | 0.063 |
| Corticospinal tract L | -4.207 | -1.597 | 0.035 |
| Medial lemniscus R | -10.121 | -4.063 | 0.300 |
| Medial lemniscus L | -9.266 | -3.720 | 0.277 |
| Inferior cerebellar peduncle R | -11.150 | -4.358 | 0.573 |
| Inferior cerebellar peduncle L | -9.301 | -3.636 | 0.439 |
| Superior cerebellar peduncle R | -12.941 | -5.176 | 0.705 |
| Superior cerebellar peduncle L | -16.208 | -6.184 | 0.635 |
| Cerebral peduncle R | -7.332 | -2.863 | 0.141 |
| Cerebral peduncle L | -7.189 | -2.451 | 0.175 |
| Anterior limb of internal capsule R | 0.000 | 0.000 | 0.000 |
| Anterior limb of internal capsule L | 0.000 | 0.000 | 0.000 |
| Posterior limb of internal capsule R | 0.000 | 0.000 | 0.000 |
| Posterior limb of internal capsule L | 0.000 | 0.000 | 0.000 |
| Retrolenticular part of internal capsule R | 0.000 | 0.000 | 0.000 |
| Retrolenticular part of internal capsule L | 0.000 | 0.000 | 0.000 |
| Anterior corona radiata R | 0.000 | 0.000 | 0.000 |
| Anterior corona radiata L | 0.000 | 0.000 | 0.000 |
| Superior corona radiata R | 0.000 | 0.000 | 0.000 |
| Superior corona radiata L | 0.000 | 0.000 | 0.000 |
| Posterior corona radiata R | 0.000 | 0.000 | 0.000 |
| Posterior corona radiata L | 0.000 | 0.000 | 0.000 |
| Posterior thalamic radiation R | 0.000 | 0.000 | 0.000 |
| Posterior thalamic radiation L | 0.000 | 0.000 | 0.000 |
| Sagittal stratum R | 0.000 | 0.000 | 0.000 |
| Sagittal stratum L | 0.000 | 0.000 | 0.000 |
| External capsule R | 0.000 | 0.000 | 0.000 |
| External capsule L | 0.000 | 0.000 | 0.000 |
| Cingulum (cingulate gyrus) R | 0.000 | 0.000 | 0.000 |
| Cingulum (cingulate gyrus) L | -3.973 | -0.998 | 0.007 |
| Cingulum (hippocampus) R | -2.973 | -1.166 | 0.021 |
| Cingulum (hippocampus) L | -3.228 | -1.053 | 0.019 |
| Fornix (cres) / Stria terminalis R | -4.362 | -1.820 | 0.131 |
| Fornix (cres) / Stria terminalis L | -4.017 | -1.431 | 0.095 |
| Superior longitudinal fasciculus R | 0.000 | 0.000 | 0.000 |
| Superior longitudinal fasciculus L | 0.000 | 0.000 | 0.000 |
| Superior fronto-occipital fasciculus R | 0.000 | 0.000 | 0.000 |
| Superior fronto-occipital fasciculus L | 0.000 | 0.000 | 0.000 |
| Uncinate fasciculus R | 0.000 | 0.000 | 0.000 |
| Uncinate fasciculus L | 0.000 | 0.000 | 0.000 |
| Tapetum R | 0.000 | 0.000 | 0.000 |
| Tapetum L | -2.946 | -0.993 | 0.001 |


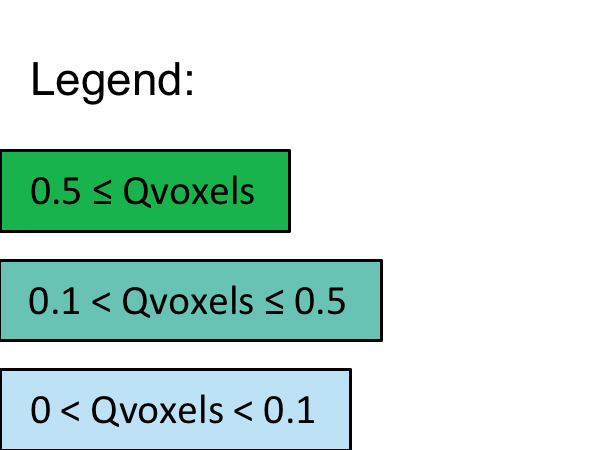


Table S6.2.1: Peak *t­* statistic value, peak Hedge’s *g* value, and the fraction of ROI voxels significantly different (FWE corrected) between HVs and SCA7 patients (Qvoxels) for the pVF group comparison within each ROI of the ICBM-DTI-81 WM atlas.

**Table S6.2.2:** pVF full FreeSurfer GM atlas table

| **ROI** | **t_peak** | **g_peak** | **Qvoxels** |
| --- | --- | --- | --- |
| Left-Cerebellum-Cortex | -15.178 | -5.707 | 0.611 |
| Left-Thalamus-Proper | -3.581 | -1.310 | 0.016 |
| Left-Caudate | 0.000 | 0.000 | 0.000 |
| Left-Putamen | 0.000 | 0.000 | 0.000 |
| Left-Pallidum | 0.000 | 0.000 | 0.000 |
| Brain-Stem | -16.208 | -6.184 | 0.219 |
| Left-Hippocampus | -4.354 | -1.692 | 0.142 |
| Left-Amygdala | -3.868 | -1.304 | 0.034 |
| Left-Accumbens-area | 0.000 | 0.000 | 0.000 |
| Left-VentralDC | -7.189 | -2.451 | 0.154 |
| Right-Cerebellum-Cortex | -10.328 | -4.036 | 0.588 |
| Right-Thalamus-Proper | -4.199 | -1.684 | 0.025 |
| Right-Caudate | 0.000 | 0.000 | 0.000 |
| Right-Putamen | 0.000 | 0.000 | 0.000 |
| Right-Pallidum | 0.000 | 0.000 | 0.000 |
| Right-Hippocampus | -4.153 | -1.646 | 0.101 |
| Right-Amygdala | -4.957 | -1.922 | 0.019 |
| Right-Accumbens-area | 0.000 | 0.000 | 0.000 |
| Right-VentralDC | -7.814 | -2.921 | 0.145 |
| ctx-lh-bankssts | 0.000 | 0.000 | 0.000 |
| ctx-lh-caudalanteriorcingulate | 0.000 | 0.000 | 0.000 |
| ctx-lh-caudalmiddlefrontal | 0.000 | 0.000 | 0.000 |
| ctx-lh-cuneus | -5.056 | -1.937 | 0.030 |
| ctx-lh-entorhinal | -4.387 | -1.572 | 0.072 |
| ctx-lh-fusiform | -5.134 | -1.603 | 0.031 |
| ctx-lh-inferiorparietal | 0.000 | 0.000 | 0.000 |
| ctx-lh-inferiortemporal | 0.000 | 0.000 | 0.000 |
| ctx-lh-isthmuscingulate | -3.973 | -1.092 | 0.029 |
| ctx-lh-lateraloccipital | -4.034 | -1.664 | 0.015 |
| ctx-lh-lateralorbitofrontal | 0.000 | 0.000 | 0.000 |
| ctx-lh-lingual | -4.056 | -1.411 | 0.003 |
| ctx-lh-medialorbitofrontal | 0.000 | 0.000 | 0.000 |
| ctx-lh-middletemporal | 0.000 | 0.000 | 0.000 |
| ctx-lh-parahippocampal | -3.321 | -1.144 | 0.014 |
| ctx-lh-paracentral | 0.000 | 0.000 | 0.000 |
| ctx-lh-parsopercularis | 0.000 | 0.000 | 0.000 |
| ctx-lh-parsorbitalis | 0.000 | 0.000 | 0.000 |
| ctx-lh-parstriangularis | 0.000 | 0.000 | 0.000 |
| ctx-lh-pericalcarine | -5.181 | -1.795 | 0.020 |
| ctx-lh-postcentral | 0.000 | 0.000 | 0.000 |
| ctx-lh-posteriorcingulate | -4.414 | -1.246 | 0.020 |
| ctx-lh-precentral | 0.000 | 0.000 | 0.000 |
| ctx-lh-precuneus | -6.004 | -1.719 | 0.003 |
| ctx-lh-rostralanteriorcingulate | 0.000 | 0.000 | 0.000 |
| ctx-lh-rostralmiddlefrontal | 0.000 | 0.000 | 0.000 |
| ctx-lh-superiorfrontal | 0.000 | 0.000 | 0.000 |
| ctx-lh-superiorparietal | 0.000 | 0.000 | 0.000 |
| ctx-lh-superiortemporal | 0.000 | 0.000 | 0.000 |
| ctx-lh-supramarginal | 0.000 | 0.000 | 0.000 |
| ctx-lh-frontalpole | 0.000 | 0.000 | 0.000 |
| ctx-lh-temporalpole | -3.951 | -1.195 | 0.027 |
| ctx-lh-transversetemporal | 0.000 | 0.000 | 0.000 |
| ctx-lh-insula | 0.000 | 0.000 | 0.000 |
| ctx-rh-bankssts | 0.000 | 0.000 | 0.000 |
| ctx-rh-caudalanteriorcingulate | 0.000 | 0.000 | 0.000 |
| ctx-rh-caudalmiddlefrontal | 0.000 | 0.000 | 0.000 |
| ctx-rh-cuneus | -6.507 | -2.545 | 0.011 |
| ctx-rh-entorhinal | -3.628 | -1.475 | 0.071 |
| ctx-rh-fusiform | -7.256 | -2.244 | 0.061 |
| ctx-rh-inferiorparietal | 0.000 | 0.000 | 0.000 |
| ctx-rh-inferiortemporal | 0.000 | 0.000 | 0.000 |
| ctx-rh-isthmuscingulate | -4.086 | -1.402 | 0.073 |
| ctx-rh-lateraloccipital | -5.636 | -2.286 | 0.021 |
| ctx-rh-lateralorbitofrontal | 0.000 | 0.000 | 0.000 |
| ctx-rh-lingual | -3.929 | -1.462 | 0.016 |
| ctx-rh-medialorbitofrontal | 0.000 | 0.000 | 0.000 |
| ctx-rh-middletemporal | 0.000 | 0.000 | 0.000 |
| ctx-rh-parahippocampal | -4.278 | -1.537 | 0.049 |
| ctx-rh-paracentral | 0.000 | 0.000 | 0.000 |
| ctx-rh-parsopercularis | 0.000 | 0.000 | 0.000 |
| ctx-rh-parsorbitalis | 0.000 | 0.000 | 0.000 |
| ctx-rh-parstriangularis | 0.000 | 0.000 | 0.000 |
| ctx-rh-pericalcarine | -4.660 | -1.797 | 0.022 |
| ctx-rh-postcentral | 0.000 | 0.000 | 0.000 |
| ctx-rh-posteriorcingulate | 0.000 | 0.000 | 0.000 |
| ctx-rh-precentral | 0.000 | 0.000 | 0.000 |
| ctx-rh-precuneus | 0.000 | 0.000 | 0.000 |
| ctx-rh-rostralanteriorcingulate | 0.000 | 0.000 | 0.000 |
| ctx-rh-rostralmiddlefrontal | 0.000 | 0.000 | 0.000 |
| ctx-rh-superiorfrontal | 0.000 | 0.000 | 0.000 |
| ctx-rh-superiorparietal | 0.000 | 0.000 | 0.000 |
| ctx-rh-superiortemporal | 0.000 | 0.000 | 0.000 |
| ctx-rh-supramarginal | 0.000 | 0.000 | 0.000 |
| ctx-rh-frontalpole | 0.000 | 0.000 | 0.000 |
| ctx-rh-temporalpole | 0.000 | 0.000 | 0.000 |
| ctx-rh-transversetemporal | 0.000 | 0.000 | 0.000 |
| ctx-rh-insula | 0.000 | 0.000 | 0.000 |


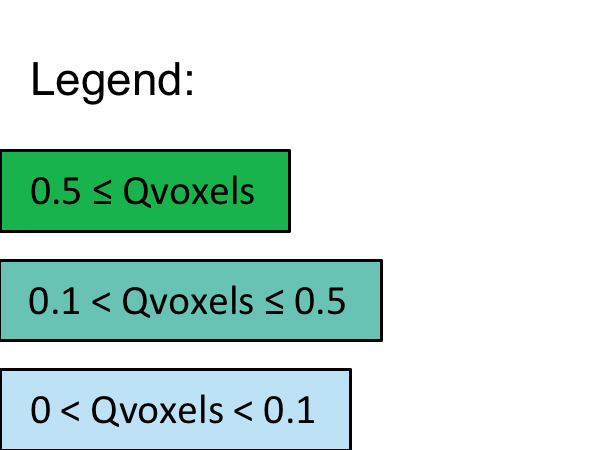


Table S6.2.2: Peak *t­* statistic value, peak Hedge’s *g* value, and the fraction of ROI voxels significantly different (FWE corrected) between HVs and SCA7 patients (Qvoxels) for the pVF group comparison within each ROI of the FreeSurfer GM atlas.

**S6.3:** pMD full tables

**Table S6.3.1:** pMD full ICBM-DTI-81 WM atlas table

| **ROI** | ***t*** peak | ***g*** peak | **Qvoxels** |
| --- | --- | --- | --- |
| Middle cerebellar peduncle | 10.373 | 4.162 | 0.608 |
| Pontine crossing tract (a part of MCP) | 5.913 | 2.404 | 0.812 |
| Genu of corpus callosum | 4.262 | 1.695 | 0.123 |
| Body of corpus callosum | 5.362 | 2.172 | 0.192 |
| Splenium of corpus callosum | 5.834 | 1.881 | 0.137 |
| Fornix (column and body of fornix) | 0.000 | 0.000 | 0.000 |
| Corticospinal tract R | 5.920 | 2.260 | 0.619 |
| Corticospinal tract L | 7.029 | 2.764 | 0.641 |
| Medial lemniscus R | 7.612 | 2.977 | 0.423 |
| Medial lemniscus L | 5.974 | 2.326 | 0.328 |
| Inferior cerebellar peduncle R | 10.308 | 4.033 | 0.297 |
| Inferior cerebellar peduncle L | 12.316 | 4.921 | 0.313 |
| Superior cerebellar peduncle R | 6.349 | 2.557 | 0.162 |
| Superior cerebellar peduncle L | 5.106 | 1.967 | 0.222 |
| Cerebral peduncle R | 5.351 | 2.161 | 0.321 |
| Cerebral peduncle L | 6.197 | 2.420 | 0.312 |
| Anterior limb of internal capsule R | 4.522 | 1.690 | 0.044 |
| Anterior limb of internal capsule L | 4.916 | 1.892 | 0.151 |
| Posterior limb of internal capsule R | 5.782 | 2.360 | 0.318 |
| Posterior limb of internal capsule L | 6.352 | 2.566 | 0.577 |
| Retrolenticular part of internal capsule R | 4.894 | 2.022 | 0.290 |
| Retrolenticular part of internal capsule L | 5.412 | 2.222 | 0.363 |
| Anterior corona radiata R | 4.970 | 1.377 | 0.135 |
| Anterior corona radiata L | 5.491 | 2.239 | 0.181 |
| Superior corona radiata R | 6.310 | 2.559 | 0.505 |
| Superior corona radiata L | 6.439 | 2.606 | 0.550 |
| Posterior corona radiata R | 4.903 | 2.014 | 0.338 |
| Posterior corona radiata L | 6.521 | 2.526 | 0.468 |
| Posterior thalamic radiation R | 6.861 | 2.763 | 0.658 |
| Posterior thalamic radiation L | 4.747 | 1.884 | 0.515 |
| Sagittal stratum R | 5.369 | 2.078 | 0.292 |
| Sagittal stratum L | 4.059 | 1.614 | 0.430 |
| External capsule R | 3.707 | 1.440 | 0.031 |
| External capsule L | 6.396 | 1.953 | 0.131 |
| Cingulum (cingulate gyrus) R | 3.164 | 1.264 | 0.048 |
| Cingulum (cingulate gyrus) L | 4.474 | 1.675 | 0.060 |
| Cingulum (hippocampus) R | 3.127 | 1.293 | 0.005 |
| Cingulum (hippocampus) L | 2.528 | 1.029 | 0.033 |
| Fornix (cres) / Stria terminalis R | 5.071 | 2.090 | 0.300 |
| Fornix (cres) / Stria terminalis L | 5.220 | 2.085 | 0.366 |
| Superior longitudinal fasciculus R | 4.969 | 1.934 | 0.306 |
| Superior longitudinal fasciculus L | 6.649 | 2.636 | 0.302 |
| Superior fronto-occipital fasciculus R | 3.682 | 1.225 | 0.163 |
| Superior fronto-occipital fasciculus L | 4.533 | 1.742 | 0.161 |
| Uncinate fasciculus R | 0.000 | 0.000 | 0.000 |
| Uncinate fasciculus L | 3.531 | 1.463 | 0.112 |
| Tapetum R | 4.788 | 1.898 | 0.236 |
| Tapetum L | 3.186 | 1.350 | 0.057 |


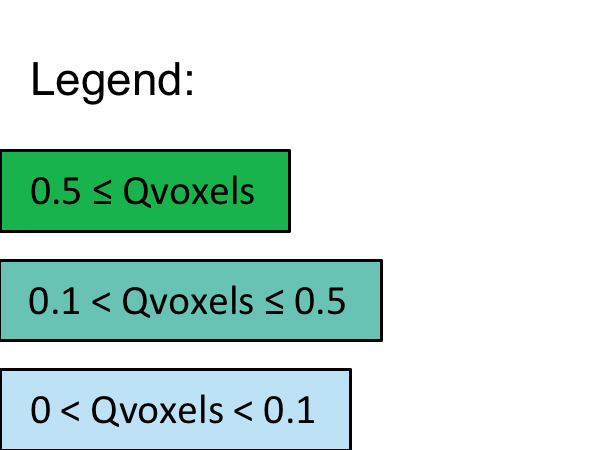


Table S6.3.1: Peak *t­* statistic value, peak Hedge’s *g* value, and the fraction of ROI voxels significantly different (FWE corrected) between HVs and SCA7 patients (Qvoxels) for the pMD group comparison within each ROI of the ICBM-DTI-81 WM atlas.

**Table S6.3.2:** pMD FreeSurfer full GM atlas table

| **ROI** | **t_peak** | **g_peak** | **Qvoxels** |
| --- | --- | --- | --- |
| Left-Cerebellum-Cortex | 6.725 | 2.534 | 0.098 |
| Left-Thalamus-Proper | 7.045 | 2.828 | 0.256 |
| Left-Caudate | 3.484 | 1.355 | 0.026 |
| Left-Putamen | 4.080 | 1.561 | 0.075 |
| Left-Pallidum | 5.079 | 1.996 | 0.185 |
| Brain-Stem | 10.308 | 4.033 | 0.408 |
| Left-Hippocampus | 3.920 | 1.512 | 0.065 |
| Left-Amygdala | 3.786 | 1.580 | 0.112 |
| Left-Accumbens-area | 0.000 | 0.000 | 0.000 |
| Left-VentralDC | 6.815 | 2.420 | 0.192 |
| Right-Cerebellum-Cortex | 9.328 | 3.629 | 0.151 |
| Right-Thalamus-Proper | 6.758 | 2.686 | 0.215 |
| Right-Caudate | 2.977 | 1.277 | 0.007 |
| Right-Putamen | 3.731 | 1.395 | 0.016 |
| Right-Pallidum | 4.541 | 1.740 | 0.177 |
| Right-Hippocampus | 2.758 | 0.990 | 0.006 |
| Right-Amygdala | 2.222 | 0.838 | 0.002 |
| Right-Accumbens-area | 0.000 | 0.000 | 0.000 |
| Right-VentralDC | 5.351 | 2.164 | 0.162 |
| ctx-lh-bankssts | 0.000 | 0.000 | 0.000 |
| ctx-lh-caudalanteriorcingulate | 3.214 | 1.185 | 0.040 |
| ctx-lh-caudalmiddlefrontal | 4.351 | 1.730 | 0.113 |
| ctx-lh-cuneus | 4.344 | 1.716 | 0.102 |
| ctx-lh-entorhinal | 3.674 | 1.556 | 0.032 |
| ctx-lh-fusiform | 4.972 | 1.862 | 0.050 |
| ctx-lh-inferiorparietal | 5.441 | 2.127 | 0.089 |
| ctx-lh-inferiortemporal | 3.796 | 1.606 | 0.034 |
| ctx-lh-isthmuscingulate | 4.183 | 1.576 | 0.013 |
| ctx-lh-lateraloccipital | 5.182 | 2.133 | 0.094 |
| ctx-lh-lateralorbitofrontal | 5.058 | 1.772 | 0.062 |
| ctx-lh-lingual | 4.556 | 1.851 | 0.091 |
| ctx-lh-medialorbitofrontal | 4.230 | 1.603 | 0.017 |
| ctx-lh-middletemporal | 4.052 | 1.591 | 0.068 |
| ctx-lh-parahippocampal | 2.930 | 1.230 | 0.009 |
| ctx-lh-paracentral | 5.010 | 1.952 | 0.073 |
| ctx-lh-parsopercularis | 4.255 | 1.488 | 0.066 |
| ctx-lh-parsorbitalis | 2.906 | 1.110 | 0.032 |
| ctx-lh-parstriangularis | 2.280 | 1.012 | 0.003 |
| ctx-lh-pericalcarine | 3.327 | 1.249 | 0.029 |
| ctx-lh-postcentral | 6.315 | 2.033 | 0.136 |
| ctx-lh-posteriorcingulate | 3.772 | 1.534 | 0.028 |
| ctx-lh-precentral | 5.175 | 1.940 | 0.139 |
| ctx-lh-precuneus | 4.548 | 1.775 | 0.109 |
| ctx-lh-rostralanteriorcingulate | 3.158 | 1.105 | 0.031 |
| ctx-lh-rostralmiddlefrontal | 4.807 | 1.789 | 0.104 |
| ctx-lh-superiorfrontal | 5.469 | 2.236 | 0.160 |
| ctx-lh-superiorparietal | 4.730 | 1.912 | 0.131 |
| ctx-lh-superiortemporal | 3.890 | 1.413 | 0.035 |
| ctx-lh-supramarginal | 4.808 | 1.990 | 0.063 |
| ctx-lh-frontalpole | 3.000 | 1.163 | 0.074 |
| ctx-lh-temporalpole | 2.446 | 1.008 | 0.002 |
| ctx-lh-transversetemporal | 3.804 | 1.423 | 0.039 |
| ctx-lh-insula | 4.353 | 1.753 | 0.030 |
| ctx-rh-bankssts | 3.171 | 1.308 | 0.026 |
| ctx-rh-caudalanteriorcingulate | 3.372 | 1.297 | 0.035 |
| ctx-rh-caudalmiddlefrontal | 4.783 | 1.755 | 0.085 |
| ctx-rh-cuneus | 3.917 | 1.508 | 0.078 |
| ctx-rh-entorhinal | 0.000 | 0.000 | 0.000 |
| ctx-rh-fusiform | 4.638 | 1.897 | 0.086 |
| ctx-rh-inferiorparietal | 5.873 | 2.124 | 0.092 |
| ctx-rh-inferiortemporal | 5.056 | 2.068 | 0.017 |
| ctx-rh-isthmuscingulate | 4.370 | 1.820 | 0.018 |
| ctx-rh-lateraloccipital | 5.069 | 1.999 | 0.124 |
| ctx-rh-lateralorbitofrontal | 0.000 | 0.000 | 0.000 |
| ctx-rh-lingual | 5.316 | 1.733 | 0.103 |
| ctx-rh-medialorbitofrontal | 0.000 | 0.000 | 0.000 |
| ctx-rh-middletemporal | 4.424 | 1.842 | 0.004 |
| ctx-rh-parahippocampal | 2.344 | 0.982 | 0.001 |
| ctx-rh-paracentral | 5.025 | 2.031 | 0.158 |
| ctx-rh-parsopercularis | 3.554 | 1.327 | 0.029 |
| ctx-rh-parsorbitalis | 0.000 | 0.000 | 0.000 |
| ctx-rh-parstriangularis | 4.156 | 1.683 | 0.095 |
| ctx-rh-pericalcarine | 5.323 | 2.163 | 0.083 |
| ctx-rh-postcentral | 5.224 | 1.881 | 0.047 |
| ctx-rh-posteriorcingulate | 4.177 | 1.669 | 0.028 |
| ctx-rh-precentral | 4.871 | 1.995 | 0.068 |
| ctx-rh-precuneus | 5.246 | 1.903 | 0.097 |
| ctx-rh-rostralanteriorcingulate | 3.333 | 1.143 | 0.008 |
| ctx-rh-rostralmiddlefrontal | 4.870 | 1.710 | 0.047 |
| ctx-rh-superiorfrontal | 4.921 | 1.980 | 0.056 |
| ctx-rh-superiorparietal | 5.431 | 2.224 | 0.109 |
| ctx-rh-superiortemporal | 3.418 | 1.430 | 0.001 |
| ctx-rh-supramarginal | 4.293 | 1.585 | 0.075 |
| ctx-rh-frontalpole | 0.000 | 0.000 | 0.000 |
| ctx-rh-temporalpole | 0.000 | 0.000 | 0.000 |
| ctx-rh-transversetemporal | 3.344 | 1.416 | 0.013 |
| ctx-rh-insula | 2.685 | 1.153 | 0.001 |


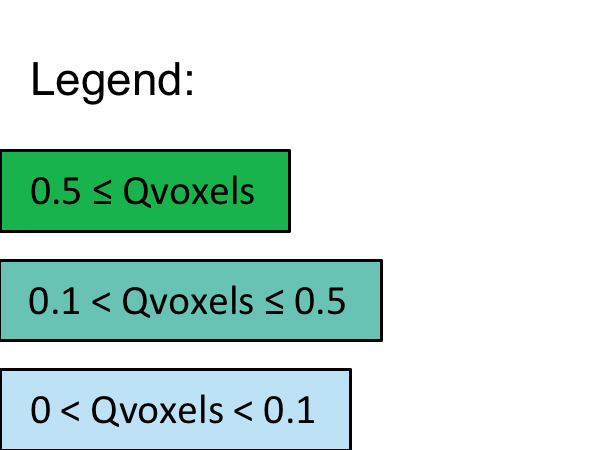


Table S6.3.2: Peak *t­* statistic value, peak Hedge’s *g* value, and the fraction of ROI voxels significantly different (FWE corrected) between HVs and SCA7 patients (Qvoxels) for the pMD group comparison within each ROI of the FreeSurfer GM atlas.

**S6.4:** pFA full tables

**Table S6.4.1:** pFA full ICBM-DTI-81 WM atlas table

| **ROI** | ***t*** peak | ***g*** peak | **Qvoxels** |
| --- | --- | --- | --- |
| Middle cerebellar peduncle | -11.768 | -4.686 | 0.550 |
| Pontine crossing tract (a part of MCP) | -7.721 | -3.116 | 0.806 |
| Genu of corpus callosum | -4.459 | -1.304 | 0.005 |
| Body of corpus callosum | -4.279 | -1.752 | 0.124 |
| Splenium of corpus callosum | -4.602 | -1.909 | 0.203 |
| Fornix (column and body of fornix) | 0.000 | 0.000 | 0.000 |
| Corticospinal tract R | -6.511 | -2.609 | 0.579 |
| Corticospinal tract L | -7.584 | -3.066 | 0.510 |
| Medial lemniscus R | -10.778 | -4.266 | 0.525 |
| Medial lemniscus L | -8.643 | -3.334 | 0.438 |
| Inferior cerebellar peduncle R | -10.071 | -3.917 | 0.311 |
| Inferior cerebellar peduncle L | -8.061 | -3.225 | 0.203 |
| Superior cerebellar peduncle R | -7.301 | -2.800 | 0.143 |
| Superior cerebellar peduncle L | -6.754 | -2.308 | 0.242 |
| Cerebral peduncle R | -6.664 | -2.698 | 0.349 |
| Cerebral peduncle L | -6.609 | -2.652 | 0.413 |
| Anterior limb of internal capsule R | -3.825 | -1.608 | 0.027 |
| Anterior limb of internal capsule L | -4.673 | -1.816 | 0.384 |
| Posterior limb of internal capsule R | -6.302 | -2.178 | 0.266 |
| Posterior limb of internal capsule L | -7.149 | -2.773 | 0.315 |
| Retrolenticular part of internal capsule R | -6.232 | -2.320 | 0.326 |
| Retrolenticular part of internal capsule L | -5.730 | -2.127 | 0.267 |
| Anterior corona radiata R | -4.064 | -1.294 | 0.021 |
| Anterior corona radiata L | -4.807 | -1.971 | 0.121 |
| Superior corona radiata R | -5.850 | -2.097 | 0.279 |
| Superior corona radiata L | -6.521 | -2.592 | 0.346 |
| Posterior corona radiata R | -5.081 | -1.926 | 0.064 |
| Posterior corona radiata L | -5.049 | -2.066 | 0.204 |
| Posterior thalamic radiation R | -7.608 | -3.032 | 0.537 |
| Posterior thalamic radiation L | -5.455 | -2.224 | 0.483 |
| Sagittal stratum R | -5.543 | -2.263 | 0.248 |
| Sagittal stratum L | -5.704 | -2.303 | 0.329 |
| External capsule R | -3.557 | -1.373 | 0.020 |
| External capsule L | -5.235 | -1.955 | 0.129 |
| Cingulum (cingulate gyrus) R | -3.634 | -1.379 | 0.023 |
| Cingulum (cingulate gyrus) L | -5.321 | -1.861 | 0.106 |
| Cingulum (hippocampus) R | -2.647 | -1.111 | 0.013 |
| Cingulum (hippocampus) L | 0.000 | 0.000 | 0.000 |
| Fornix (cres) / Stria terminalis R | -3.917 | -1.649 | 0.212 |
| Fornix (cres) / Stria terminalis L | -6.761 | -2.744 | 0.359 |
| Superior longitudinal fasciculus R | -6.822 | -1.682 | 0.161 |
| Superior longitudinal fasciculus L | -4.146 | -1.670 | 0.091 |
| Superior fronto-occipital fasciculus R | -3.102 | -0.991 | 0.107 |
| Superior fronto-occipital fasciculus L | -4.162 | -1.684 | 0.085 |
| Uncinate fasciculus R | 0.000 | 0.000 | 0.000 |
| Uncinate fasciculus L | 0.000 | 0.000 | 0.000 |
| Tapetum R | -3.938 | -1.589 | 0.249 |
| Tapetum L | -4.678 | -1.936 | 0.182 |


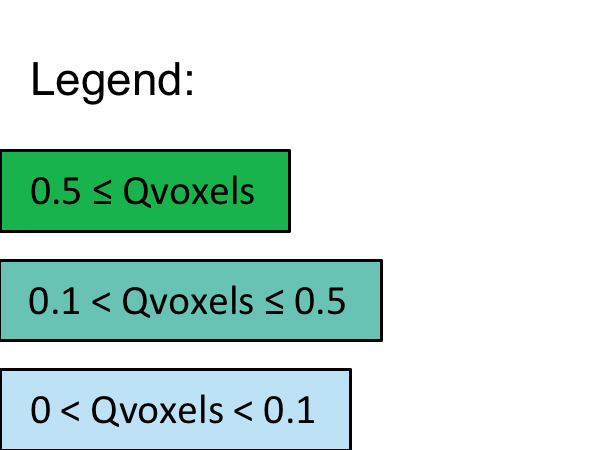


Table S6.4.1: Peak *t­* statistic value, peak Hedge’s *g* value, and the fraction of ROI voxels significantly different (FWE corrected) between HVs and SCA7 patients (Qvoxels) for the pFA group comparison within each ROI of the ICBM-DTI-81 WM atlas.

**Table S6.4.2:** pFA full FreeSurfer GM atlas table

| **ROI** | **t_peak** | **g_peak** | **Qvoxels** |
| --- | --- | --- | --- |
| Left-Cerebellum-Cortex | -9.739 | -3.789 | 0.548 |
| Left-Thalamus-Proper | -8.241 | -3.322 | 0.168 |
| Left-Caudate | -3.410 | -1.418 | 0.064 |
| Left-Putamen | -3.937 | -1.536 | 0.163 |
| Left-Pallidum | -6.079 | -2.372 | 0.180 |
| Brain-Stem | -10.778 | -4.266 | 0.370 |
| Left-Hippocampus | -3.060 | -1.211 | 0.009 |
| Left-Amygdala | -2.543 | -1.130 | 0.026 |
| Left-Accumbens-area | 0.000 | 0.000 | 0.000 |
| Left-VentralDC | -6.330 | -2.553 | 0.302 |
| Right-Cerebellum-Cortex | -10.831 | -4.254 | 0.608 |
| Right-Thalamus-Proper | -5.730 | -2.320 | 0.176 |
| Right-Caudate | -2.474 | -0.929 | 0.002 |
| Right-Putamen | -4.428 | -1.701 | 0.030 |
| Right-Pallidum | -5.333 | -2.144 | 0.183 |
| Right-Hippocampus | 0.000 | 0.000 | 0.000 |
| Right-Amygdala | 0.000 | 0.000 | 0.000 |
| Right-Accumbens-area | 0.000 | 0.000 | 0.000 |
| Right-VentralDC | -5.189 | -2.136 | 0.186 |


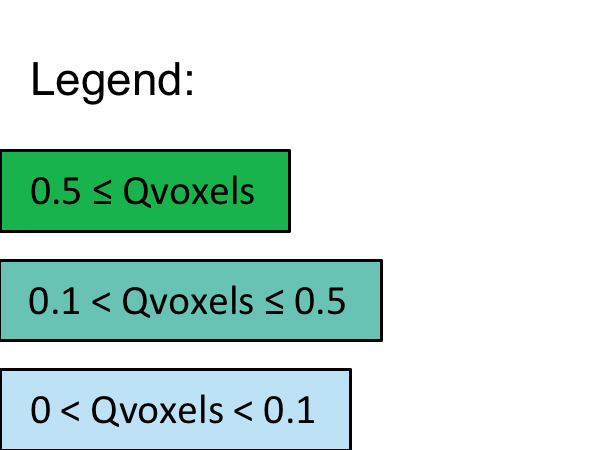


Table S6.4.2: Peak *t­* statistic value, peak Hedge’s *g* value, and the fraction of ROI voxels significantly different (FWE corrected) between HVs and SCA7 patients (Qvoxels) for the pFA group comparison within each ROI of the FreeSurfer GM atlas.

**S6.5:** VBM full table

**Table S6.5.1:** VBM full FreeSurfer GM atlas table

| **ROI** | **t_peak** | **g_peak** | **Qvoxels** |
| --- | --- | --- | --- |
| Left-Cerebellum-Cortex | -11.242 | -4.199 | 0.282 |
| Left-Thalamus-Proper | -8.238 | -2.122 | 0.199 |
| Left-Caudate | -4.067 | -1.721 | 0.018 |
| Left-Putamen | -4.310 | -1.790 | 0.015 |
| Left-Pallidum | -4.453 | -1.647 | 0.030 |
| Brain-Stem | -12.066 | -4.570 | 0.794 |
| Left-Hippocampus | -3.084 | -0.988 | 0.007 |
| Left-Amygdala | 0.000 | 0.000 | 0.000 |
| Left-Accumbens-area | 0.000 | 0.000 | 0.000 |
| Left-VentralDC | -6.790 | -2.338 | 0.177 |
| Right-Cerebellum-Cortex | -10.527 | -2.847 | 0.243 |
| Right-Thalamus-Proper | -7.765 | -3.067 | 0.259 |
| Right-Caudate | -5.035 | -2.323 | 0.079 |
| Right-Putamen | -5.790 | -2.092 | 0.078 |
| Right-Pallidum | -5.851 | -2.170 | 0.080 |
| Right-Hippocampus | -3.486 | -1.171 | 0.020 |
| Right-Amygdala | 0.000 | 0.000 | 0.000 |
| Right-Accumbens-area | 0.000 | 0.000 | 0.000 |
| Right-VentralDC | -6.782 | -2.270 | 0.234 |
| ctx-lh-bankssts | 0.000 | 0.000 | 0.000 |
| ctx-lh-caudalanteriorcingulate | 0.000 | 0.000 | 0.000 |
| ctx-lh-caudalmiddlefrontal | 0.000 | 0.000 | 0.000 |
| ctx-lh-cuneus | 0.000 | 0.000 | 0.000 |
| ctx-lh-entorhinal | 0.000 | 0.000 | 0.000 |
| ctx-lh-fusiform | -5.154 | -2.174 | 0.054 |
| ctx-lh-inferiorparietal | 0.000 | 0.000 | 0.000 |
| ctx-lh-inferiortemporal | 0.000 | 0.000 | 0.000 |
| ctx-lh-isthmuscingulate | -2.966 | -0.794 | 0.005 |
| ctx-lh-lateraloccipital | 0.000 | 0.000 | 0.000 |
| ctx-lh-lateralorbitofrontal | 0.000 | 0.000 | 0.000 |
| ctx-lh-lingual | -4.064 | -1.559 | 0.089 |
| ctx-lh-medialorbitofrontal | 0.000 | 0.000 | 0.000 |
| ctx-lh-middletemporal | 0.000 | 0.000 | 0.000 |
| ctx-lh-parahippocampal | 0.000 | 0.000 | 0.000 |
| ctx-lh-paracentral | 0.000 | 0.000 | 0.000 |
| ctx-lh-parsopercularis | 0.000 | 0.000 | 0.000 |
| ctx-lh-parsorbitalis | 0.000 | 0.000 | 0.000 |
| ctx-lh-parstriangularis | 0.000 | 0.000 | 0.000 |
| ctx-lh-pericalcarine | 0.000 | 0.000 | 0.000 |
| ctx-lh-postcentral | -3.670 | -0.961 | 0.001 |
| ctx-lh-posteriorcingulate | 0.000 | 0.000 | 0.000 |
| ctx-lh-precentral | -4.331 | -0.936 | 0.021 |
| ctx-lh-precuneus | 0.000 | 0.000 | 0.000 |
| ctx-lh-rostralanteriorcingulate | 0.000 | 0.000 | 0.000 |
| ctx-lh-rostralmiddlefrontal | 0.000 | 0.000 | 0.000 |
| ctx-lh-superiorfrontal | 0.000 | 0.000 | 0.000 |
| ctx-lh-superiorparietal | -3.756 | -1.009 | 0.001 |
| ctx-lh-superiortemporal | 0.000 | 0.000 | 0.000 |
| ctx-lh-supramarginal | 0.000 | 0.000 | 0.000 |
| ctx-lh-frontalpole | 0.000 | 0.000 | 0.000 |
| ctx-lh-temporalpole | 0.000 | 0.000 | 0.000 |
| ctx-lh-transversetemporal | 0.000 | 0.000 | 0.000 |
| ctx-lh-insula | 0.000 | 0.000 | 0.000 |
| ctx-rh-bankssts | 0.000 | 0.000 | 0.000 |
| ctx-rh-caudalanteriorcingulate | 0.000 | 0.000 | 0.000 |
| ctx-rh-caudalmiddlefrontal | 0.000 | 0.000 | 0.000 |
| ctx-rh-cuneus | 0.000 | 0.000 | 0.000 |
| ctx-rh-entorhinal | -2.821 | -1.026 | 0.003 |
| ctx-rh-fusiform | -6.412 | -2.493 | 0.069 |
| ctx-rh-inferiorparietal | 0.000 | 0.000 | 0.000 |
| ctx-rh-inferiortemporal | 0.000 | 0.000 | 0.000 |
| ctx-rh-isthmuscingulate | 0.000 | 0.000 | 0.000 |
| ctx-rh-lateraloccipital | 0.000 | 0.000 | 0.000 |
| ctx-rh-lateralorbitofrontal | 0.000 | 0.000 | 0.000 |
| ctx-rh-lingual | -4.574 | -1.618 | 0.036 |
| ctx-rh-medialorbitofrontal | 0.000 | 0.000 | 0.000 |
| ctx-rh-middletemporal | 0.000 | 0.000 | 0.000 |
| ctx-rh-parahippocampal | 0.000 | 0.000 | 0.000 |
| ctx-rh-paracentral | 0.000 | 0.000 | 0.000 |
| ctx-rh-parsopercularis | 0.000 | 0.000 | 0.000 |
| ctx-rh-parsorbitalis | 0.000 | 0.000 | 0.000 |
| ctx-rh-parstriangularis | 0.000 | 0.000 | 0.000 |
| ctx-rh-pericalcarine | 0.000 | 0.000 | 0.000 |
| ctx-rh-postcentral | -3.905 | -1.344 | 0.053 |
| ctx-rh-posteriorcingulate | -2.881 | -0.778 | 0.004 |
| ctx-rh-precentral | -6.153 | -1.760 | 0.107 |
| ctx-rh-precuneus | 0.000 | 0.000 | 0.000 |
| ctx-rh-rostralanteriorcingulate | 0.000 | 0.000 | 0.000 |
| ctx-rh-rostralmiddlefrontal | 0.000 | 0.000 | 0.000 |
| ctx-rh-superiorfrontal | 0.000 | 0.000 | 0.000 |
| ctx-rh-superiorparietal | -4.216 | -1.341 | 0.020 |
| ctx-rh-superiortemporal | 0.000 | 0.000 | 0.000 |
| ctx-rh-supramarginal | -3.550 | -1.208 | 0.011 |
| ctx-rh-frontalpole | 0.000 | 0.000 | 0.000 |
| ctx-rh-temporalpole | 0.000 | 0.000 | 0.000 |
| ctx-rh-transversetemporal | 0.000 | 0.000 | 0.000 |
| ctx-rh-insula | 0.000 | 0.000 | 0.000 |


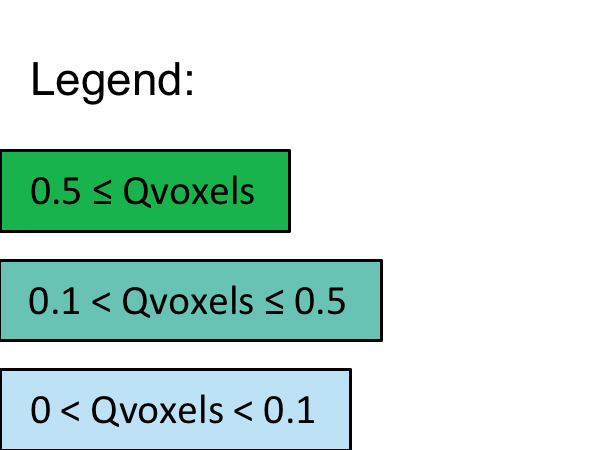


Table S6.5.1: Peak *t­* statistic value, peak Hedge’s *g* value, and the fraction of ROI voxels significantly different (FWE corrected) between HVs and SCA7 patients (Qvoxels) for the VBM group comparison within each ROI of the FreeSurfer GM atlas.

**S6.6:** MD full tables

**Table S6.6.1:** MD full ICBM-DTI-81 WM atlas table

| **ROI** | ***t*** peak | ***g*** peak | **Qvoxels** |
| --- | --- | --- | --- |
| Middle cerebellar peduncle | 11.879 | 4.706 | 0.922 |
| Pontine crossing tract (a part of MCP) | 11.588 | 4.443 | 0.960 |
| Genu of corpus callosum | 5.617 | 1.825 | 0.074 |
| Body of corpus callosum | 4.942 | 1.703 | 0.196 |
| Splenium of corpus callosum | 4.803 | 1.687 | 0.079 |
| Fornix (column and body of fornix) | 3.251 | 1.088 | 0.023 |
| Corticospinal tract R | 7.170 | 2.889 | 0.786 |
| Corticospinal tract L | 7.453 | 2.918 | 0.812 |
| Medial lemniscus R | 11.524 | 4.396 | 0.773 |
| Medial lemniscus L | 10.868 | 3.923 | 0.781 |
| Inferior cerebellar peduncle R | 11.249 | 4.508 | 0.903 |
| Inferior cerebellar peduncle L | 10.490 | 4.042 | 0.813 |
| Superior cerebellar peduncle R | 14.568 | 5.362 | 0.896 |
| Superior cerebellar peduncle L | 11.237 | 4.472 | 0.838 |
| Cerebral peduncle R | 7.082 | 2.862 | 0.538 |
| Cerebral peduncle L | 8.495 | 2.989 | 0.538 |
| Anterior limb of internal capsule R | 6.560 | 2.608 | 0.010 |
| Anterior limb of internal capsule L | 6.849 | 2.725 | 0.342 |
| Posterior limb of internal capsule R | 6.621 | 2.688 | 0.647 |
| Posterior limb of internal capsule L | 8.300 | 3.254 | 0.772 |
| Retrolenticular part of internal capsule R | 5.996 | 2.429 | 0.369 |
| Retrolenticular part of internal capsule L | 6.082 | 2.457 | 0.330 |
| Anterior corona radiata R | 3.724 | 1.319 | 0.025 |
| Anterior corona radiata L | 4.343 | 1.607 | 0.110 |
| Superior corona radiata R | 6.012 | 2.440 | 0.356 |
| Superior corona radiata L | 6.240 | 2.372 | 0.735 |
| Posterior corona radiata R | 4.441 | 1.833 | 0.280 |
| Posterior corona radiata L | 4.918 | 1.982 | 0.424 |
| Posterior thalamic radiation R | 4.221 | 1.759 | 0.211 |
| Posterior thalamic radiation L | 4.808 | 1.854 | 0.290 |
| Sagittal stratum R | 3.957 | 1.587 | 0.239 |
| Sagittal stratum L | 4.260 | 1.773 | 0.449 |
| External capsule R | 2.847 | 1.228 | 0.004 |
| External capsule L | 5.381 | 2.086 | 0.181 |
| Cingulum (cingulate gyrus) R | 4.087 | 1.627 | 0.148 |
| Cingulum (cingulate gyrus) L | 5.324 | 1.946 | 0.299 |
| Cingulum (hippocampus) R | 4.182 | 1.697 | 0.199 |
| Cingulum (hippocampus) L | 3.332 | 1.004 | 0.042 |
| Fornix (cres) / Stria terminalis R | 5.883 | 2.396 | 0.696 |
| Fornix (cres) / Stria terminalis L | 7.055 | 2.816 | 0.669 |
| Superior longitudinal fasciculus R | 3.995 | 1.620 | 0.115 |
| Superior longitudinal fasciculus L | 4.862 | 1.990 | 0.331 |
| Superior fronto-occipital fasciculus R | 3.528 | 1.497 | 0.110 |
| Superior fronto-occipital fasciculus L | 3.707 | 1.509 | 0.268 |
| Uncinate fasciculus R | 0.000 | 0.000 | 0.000 |
| Uncinate fasciculus L | 5.660 | 2.171 | 0.378 |
| Tapetum R | 4.221 | 1.605 | 0.090 |
| Tapetum L | 3.335 | 1.282 | 0.020 |


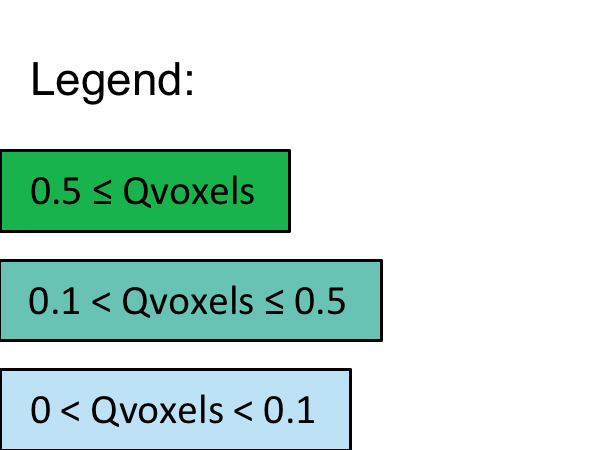


Table S6.6.1: Peak *t­* statistic value, peak Hedge’s *g* value, and the fraction of ROI voxels significantly different (FWE corrected) between HVs and SCA7 patients (Qvoxels) for the MD group comparison within each ROI of the ICBM-DTI-81 WM atlas.

**Table S6.6.2:** MD full FreeSurfer GM atlas table

| **ROI** | **t_peak** | **g_peak** | **Qvoxels** |
| --- | --- | --- | --- |
| Left-Cerebellum-Cortex | 11.504 | 4.558 | 0.807 |
| Left-Thalamus-Proper | 8.300 | 3.254 | 0.454 |
| Left-Caudate | 5.007 | 1.820 | 0.066 |
| Left-Putamen | 5.534 | 2.100 | 0.127 |
| Left-Pallidum | 4.688 | 1.939 | 0.103 |
| Brain-Stem | 14.568 | 5.362 | 0.742 |
| Left-Hippocampus | 5.023 | 1.876 | 0.192 |
| Left-Amygdala | 4.554 | 1.641 | 0.115 |
| Left-Accumbens-area | 0.000 | 0.000 | 0.000 |
| Left-VentralDC | 7.482 | 2.965 | 0.442 |
| Right-Cerebellum-Cortex | 11.628 | 4.418 | 0.815 |
| Right-Thalamus-Proper | 9.102 | 3.659 | 0.463 |
| Right-Caudate | 4.366 | 1.414 | 0.027 |
| Right-Putamen | 4.538 | 1.879 | 0.014 |
| Right-Pallidum | 4.457 | 1.778 | 0.123 |
| Right-Hippocampus | 4.651 | 1.847 | 0.154 |
| Right-Amygdala | 2.422 | 1.014 | 0.004 |
| Right-Accumbens-area | 0.000 | 0.000 | 0.000 |
| Right-VentralDC | 7.944 | 3.033 | 0.417 |
| ctx-lh-bankssts | 3.092 | 1.285 | 0.002 |
| ctx-lh-caudalanteriorcingulate | 4.777 | 1.654 | 0.262 |
| ctx-lh-caudalmiddlefrontal | 4.258 | 1.762 | 0.065 |
| ctx-lh-cuneus | 5.405 | 2.072 | 0.161 |
| ctx-lh-entorhinal | 4.981 | 1.967 | 0.210 |
| ctx-lh-fusiform | 5.619 | 2.188 | 0.129 |
| ctx-lh-inferiorparietal | 4.305 | 1.351 | 0.026 |
| ctx-lh-inferiortemporal | 4.134 | 1.597 | 0.051 |
| ctx-lh-isthmuscingulate | 4.514 | 1.517 | 0.134 |
| ctx-lh-lateraloccipital | 5.871 | 2.003 | 0.139 |
| ctx-lh-lateralorbitofrontal | 5.454 | 1.898 | 0.004 |
| ctx-lh-lingual | 5.089 | 2.079 | 0.159 |
| ctx-lh-medialorbitofrontal | 2.272 | 0.903 | 0.001 |
| ctx-lh-middletemporal | 3.388 | 1.419 | 0.006 |
| ctx-lh-parahippocampal | 5.180 | 1.988 | 0.074 |
| ctx-lh-paracentral | 4.604 | 1.594 | 0.117 |
| ctx-lh-parsopercularis | 4.748 | 1.785 | 0.080 |
| ctx-lh-parsorbitalis | 0.000 | 0.000 | 0.000 |
| ctx-lh-parstriangularis | 3.133 | 1.236 | 0.003 |
| ctx-lh-pericalcarine | 4.846 | 1.801 | 0.358 |
| ctx-lh-postcentral | 5.091 | 1.735 | 0.119 |
| ctx-lh-posteriorcingulate | 4.942 | 1.611 | 0.194 |
| ctx-lh-precentral | 5.123 | 1.975 | 0.089 |
| ctx-lh-precuneus | 6.319 | 1.701 | 0.123 |
| ctx-lh-rostralanteriorcingulate | 4.319 | 1.595 | 0.051 |
| ctx-lh-rostralmiddlefrontal | 5.234 | 1.827 | 0.039 |
| ctx-lh-superiorfrontal | 4.986 | 1.805 | 0.063 |
| ctx-lh-superiorparietal | 6.344 | 2.553 | 0.075 |
| ctx-lh-superiortemporal | 4.285 | 1.410 | 0.014 |
| ctx-lh-supramarginal | 4.123 | 1.603 | 0.027 |
| ctx-lh-frontalpole | 0.000 | 0.000 | 0.000 |
| ctx-lh-temporalpole | 3.692 | 1.317 | 0.034 |
| ctx-lh-transversetemporal | 3.464 | 1.318 | 0.011 |
| ctx-lh-insula | 5.865 | 2.171 | 0.147 |
| ctx-rh-bankssts | 3.669 | 1.096 | 0.016 |
| ctx-rh-caudalanteriorcingulate | 4.283 | 1.631 | 0.182 |
| ctx-rh-caudalmiddlefrontal | 3.539 | 1.100 | 0.014 |
| ctx-rh-cuneus | 5.312 | 2.094 | 0.197 |
| ctx-rh-entorhinal | 5.138 | 2.027 | 0.065 |
| ctx-rh-fusiform | 7.262 | 2.575 | 0.163 |
| ctx-rh-inferiorparietal | 4.354 | 1.478 | 0.083 |
| ctx-rh-inferiortemporal | 3.859 | 1.614 | 0.024 |
| ctx-rh-isthmuscingulate | 4.400 | 1.595 | 0.226 |
| ctx-rh-lateraloccipital | 5.761 | 2.188 | 0.233 |
| ctx-rh-lateralorbitofrontal | 0.000 | 0.000 | 0.000 |
| ctx-rh-lingual | 6.828 | 1.905 | 0.137 |
| ctx-rh-medialorbitofrontal | 0.000 | 0.000 | 0.000 |
| ctx-rh-middletemporal | 4.813 | 1.992 | 0.037 |
| ctx-rh-parahippocampal | 5.229 | 2.113 | 0.185 |
| ctx-rh-paracentral | 7.162 | 2.837 | 0.217 |
| ctx-rh-parsopercularis | 4.350 | 1.579 | 0.002 |
| ctx-rh-parsorbitalis | 0.000 | 0.000 | 0.000 |
| ctx-rh-parstriangularis | 4.350 | 1.615 | 0.001 |
| ctx-rh-pericalcarine | 6.305 | 2.230 | 0.347 |
| ctx-rh-postcentral | 4.085 | 1.450 | 0.064 |
| ctx-rh-posteriorcingulate | 5.867 | 1.645 | 0.228 |
| ctx-rh-precentral | 4.619 | 1.631 | 0.062 |
| ctx-rh-precuneus | 5.443 | 1.898 | 0.087 |
| ctx-rh-rostralanteriorcingulate | 2.911 | 1.070 | 0.005 |
| ctx-rh-rostralmiddlefrontal | 2.215 | 0.902 | 0.000 |
| ctx-rh-superiorfrontal | 4.933 | 1.831 | 0.057 |
| ctx-rh-superiorparietal | 5.419 | 1.970 | 0.079 |
| ctx-rh-superiortemporal | 3.487 | 1.213 | 0.011 |
| ctx-rh-supramarginal | 5.342 | 1.890 | 0.033 |
| ctx-rh-frontalpole | 0.000 | 0.000 | 0.000 |
| ctx-rh-temporalpole | 0.000 | 0.000 | 0.000 |
| ctx-rh-transversetemporal | 4.025 | 1.551 | 0.064 |
| ctx-rh-insula | 5.401 | 1.783 | 0.021 |


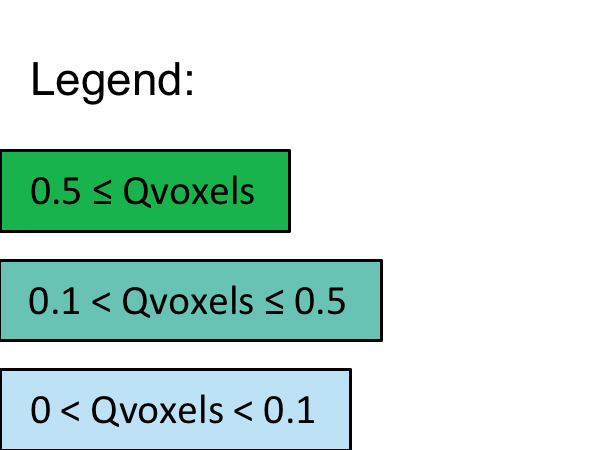


Table S6.6.2: Peak *t­* statistic value, peak Hedge’s *g* value, and the fraction of ROI voxels significantly different (FWE corrected) between HVs and SCA7 patients (Qvoxels) for the MD group comparison within each ROI of the FreeSurfer GM atlas.

**S6.7:** FA full tables

**Table S6.7.1:** FA full ICBM-DTI-81 WM atlas table

| **ROI** | ***t*** peak | ***g*** peak | **Qvoxels** |
| --- | --- | --- | --- |
| Middle cerebellar peduncle | -14.007 | -4.536 | 0.740 |
| Pontine crossing tract (a part of MCP) | -7.921 | -2.995 | 0.910 |
| Genu of corpus callosum | -4.197 | -1.570 | 0.058 |
| Body of corpus callosum | -4.918 | -1.702 | 0.145 |
| Splenium of corpus callosum | -4.652 | -1.872 | 0.163 |
| Fornix (column and body of fornix) | -2.682 | -0.900 | 0.010 |
| Corticospinal tract R | -6.023 | -2.445 | 0.675 |
| Corticospinal tract L | -7.951 | -2.969 | 0.626 |
| Medial lemniscus R | -10.257 | -3.596 | 0.884 |
| Medial lemniscus L | -10.361 | -3.718 | 0.819 |
| Inferior cerebellar peduncle R | -13.507 | -4.956 | 0.671 |
| Inferior cerebellar peduncle L | -12.553 | -5.002 | 0.515 |
| Superior cerebellar peduncle R | -16.047 | -5.455 | 0.929 |
| Superior cerebellar peduncle L | -16.335 | -5.591 | 0.892 |
| Cerebral peduncle R | -7.620 | -2.961 | 0.456 |
| Cerebral peduncle L | -8.831 | -3.276 | 0.603 |
| Anterior limb of internal capsule R | -3.985 | -1.652 | 0.004 |
| Anterior limb of internal capsule L | -6.877 | -2.755 | 0.443 |
| Posterior limb of internal capsule R | -6.265 | -2.436 | 0.268 |
| Posterior limb of internal capsule L | -6.649 | -2.342 | 0.297 |
| Retrolenticular part of internal capsule R | -5.508 | -1.831 | 0.411 |
| Retrolenticular part of internal capsule L | -5.227 | -1.914 | 0.196 |
| Anterior corona radiata R | -5.664 | -1.745 | 0.004 |
| Anterior corona radiata L | -5.115 | -1.628 | 0.134 |
| Superior corona radiata R | -4.913 | -1.915 | 0.103 |
| Superior corona radiata L | -5.299 | -2.115 | 0.282 |
| Posterior corona radiata R | -3.843 | -1.598 | 0.037 |
| Posterior corona radiata L | -3.834 | -1.617 | 0.089 |
| Posterior thalamic radiation R | -4.702 | -1.945 | 0.432 |
| Posterior thalamic radiation L | -5.519 | -2.250 | 0.378 |
| Sagittal stratum R | -4.356 | -1.790 | 0.193 |
| Sagittal stratum L | -4.704 | -1.945 | 0.276 |
| External capsule R | -3.326 | -1.270 | 0.014 |
| External capsule L | -5.727 | -2.021 | 0.087 |
| Cingulum (cingulate gyrus) R | -3.267 | -1.312 | 0.007 |
| Cingulum (cingulate gyrus) L | -4.081 | -1.652 | 0.097 |
| Cingulum (hippocampus) R | -3.700 | -1.470 | 0.030 |
| Cingulum (hippocampus) L | 0.000 | 0.000 | 0.000 |
| Fornix (cres) / Stria terminalis R | -7.278 | -2.946 | 0.553 |
| Fornix (cres) / Stria terminalis L | -7.380 | -2.972 | 0.574 |
| Superior longitudinal fasciculus R | -3.665 | -1.127 | 0.010 |
| Superior longitudinal fasciculus L | -4.187 | -1.755 | 0.038 |
| Superior fronto-occipital fasciculus R | 0.000 | 0.000 | 0.000 |
| Superior fronto-occipital fasciculus L | -2.846 | -1.053 | 0.044 |
| Uncinate fasciculus R | 0.000 | 0.000 | 0.000 |
| Uncinate fasciculus L | 0.000 | 0.000 | 0.000 |
| Tapetum R | -3.923 | -1.621 | 0.288 |
| Tapetum L | -4.273 | -1.713 | 0.282 |


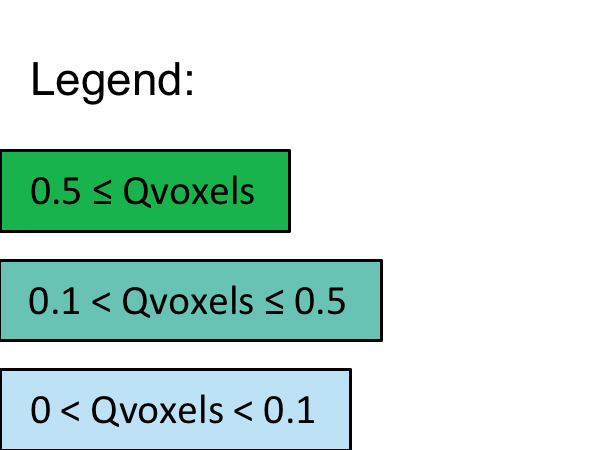


Table S6.7.1: Peak *t­* statistic value, peak Hedge’s *g* value, and the fraction of ROI voxels significantly different (FWE corrected) between HVs and SCA7 patients (Qvoxels) for the FA group comparison within each ROI of the ICBM-DTI-81 WM atlas.

**Table S6.7.2:** FA full FreeSurfer GM atlas table

| **ROI** | **t_peak** | **g_peak** | **Qvoxels** |
| --- | --- | --- | --- |
| Left-Cerebellum-Cortex | -15.155 | -5.471 | 0.829 |
| Left-Thalamus-Proper | -6.382 | -2.560 | 0.276 |
| Left-Caudate | -4.602 | -1.833 | 0.136 |
| Left-Putamen | -4.722 | -1.881 | 0.127 |
| Left-Pallidum | -5.069 | -1.991 | 0.184 |
| Brain-Stem | -16.335 | -5.591 | 0.639 |
| Left-Hippocampus | -7.866 | -3.114 | 0.350 |
| Left-Amygdala | -6.477 | -2.105 | 0.118 |
| Left-Accumbens-area | 0.000 | 0.000 | 0.000 |
| Left-VentralDC | -8.732 | -3.276 | 0.524 |
| Right-Cerebellum-Cortex | -15.866 | -5.266 | 0.824 |
| Right-Thalamus-Proper | -6.969 | -2.816 | 0.261 |
| Right-Caudate | -2.263 | -1.039 | 0.002 |
| Right-Putamen | -4.328 | -1.788 | 0.029 |
| Right-Pallidum | -6.381 | -2.464 | 0.180 |
| Right-Hippocampus | -5.964 | -2.356 | 0.287 |
| Right-Amygdala | -3.476 | -1.345 | 0.051 |
| Right-Accumbens-area | 0.000 | 0.000 | 0.000 |
| Right-VentralDC | -7.614 | -2.925 | 0.435 |


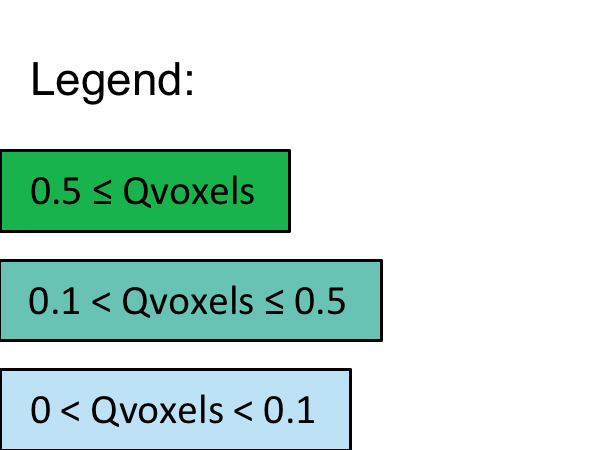


Table S6.7.2: Peak *t­* statistic value, peak Hedge’s *g* value, and the fraction of ROI voxels significantly different (FWE corrected) between HVs and SCA7 patients (Qvoxels) for the FA group comparison within each ROI of the FreeSurfer GM atlas.
